# Understanding the Role of Solvents in Hypromellose-Based Film-Forming Solutions for Topical Administration of Ketoprofen Through a Design of Experiments Approach

**DOI:** 10.3390/pharmaceutics18101249

**Published:** 2026-10-01

**Authors:** Sandra Milinković, Ivana Kurćubić, Jelena Đuriš, Ljiljana Đekić

**Affiliations:** Department of Pharmaceutical Technology and Cosmetology, Faculty of Pharmacy, University of Belgrade, Vojvode Stepe 450, 11221 Belgrade, Serbia; ivana.kurcubic@pharmacy.bg.ac.rs (I.K.); jelena.djuris@pharmacy.bg.ac.rs (J.Đ.); ljiljana.djekic@pharmacy.bg.ac.rs (L.Đ.)

**Keywords:** film-forming solution, hypromellose, ketoprofen, design of experiments, full factorial design, mechanical properties, in vitro drug release

## Abstract

**Background/Objectives**: Topical ketoprofen gels are limited by the need for frequent reapplication, short skin residence time, and the risk of sunlight-induced photoallergic dermatitis. Film-forming solutions (FFSs) allow for extended retention; however, the solvents used may affect the polymer film’s characteristics. The present study aimed to elucidate the effect of macrogol 400 and isopropanol and develop a hypromellose-based ketoprofen FFS using a design of experiments (DoE) approach. **Methods**: A 3^2^ full factorial design was applied to investigate the effects of macrogol 400 (0–5%, *w*/*w*) (X_1_) and isopropanol (25–50%, *w*/*w*) concentrations (X_2_) on film mechanical properties (tensile strength (Y_1_), elongation at break (Y_2_), and Young’s modulus (Y_3_)). The DoE-based placebo and ketoprofen-loaded FFSs were characterized for their physicochemical, rheological, and textural properties, while the corresponding films were evaluated for appearance, weight, stickiness, and mechanical properties. In vitro ketoprofen release from the drug-loaded FFS was also evaluated. **Results**: Macrogol 400 concentration was identified as a significant factor influencing film mechanical properties. As a film plasticizer, macrogol 400 improved film mechanical properties up to the optimal concentration (2.75%), whereas the water/isopropanol ratio mainly affected the applicative (rheological and textural) properties of the FFSs. Ketoprofen incorporation significantly reduced the film tensile strength and elongation at break. In vitro release testing demonstrated 58.52% ketoprofen release after 6 h, with the Weibull model showing the best fit to the release profile. **Conclusions**: Macrogol 400 and isopropanol have a complex, mutually conditioned influence on the application characteristics of hypromellose-based FFS and the mechanical characteristics of the films. Incorporation of ketoprofen into the selected placebo formulation significantly reduced film deformability but yielded a thin, transparent film with favorable in vitro drug-release characteristics. The developed FFS represents a promising topical dosage form for non-steroidal anti-inflammatory drugs and warrants further investigation, particularly in terms of skin retention and in vivo performance.

## 1. Introduction

Ketoprofen, a propionic acid derivative belonging to the class of non-steroidal anti-inflammatory drugs (NSAIDs), is widely used for the treatment of pain and inflammation associated with acute and chronic musculoskeletal disorders, including conditions such as osteoarthritis and soft-tissue injuries [1]. Its low molecular weight (254.28 g/mol) and lipophilic character (logP 3.12) [2] are considered favorable for percutaneous delivery, as these properties may facilitate partitioning into the stratum corneum and subsequent diffusion through the skin. These physicochemical characteristics make ketoprofen well suited for topical administration, which may be particularly advantageous for NSAIDs, as reducing systemic drug exposure can potentially decrease the risk of adverse effects associated with their oral administration, such as gastrointestinal discomfort and ulceration [1]. On the drug market, the most common topical dosage form of ketoprofen is gel. However, the efficacy of such conventional semisolid topical formulations may be influenced by several factors, including the need for repeated application to maintain therapeutic drug levels, the drug removal from the site of application or displacement due to contact with clothing or other surfaces, and the risk of sunlight-induced photoallergic dermatitis, potentially exacerbated by the transfer of ketoprofen to other skin regions [3,4]. These limitations have stimulated the development of advanced topical drug delivery systems capable of improving drug retention on the skin. In this context, film-forming solutions (FFSs) represent promising carriers, as they are intended for application to the skin in a liquid state and subsequently form a thin film that adheres closely to the skin following volatile solvent evaporation [3,5,6]. The liquid nature of FFSs may facilitate uniform spreading over the skin and enable convenient administration of a defined dose over the target area. In addition, the in situ formation of the film may provide several advantages, including prolonged drug residence time at the application site, reduced susceptibility to removal by contact, and a potentially improved drug-release profile based on increased thermodynamic activity due to formulation metamorphosis [3].

FFSs typically contain drug substance and one or more film-forming polymers dissolved in a mixture of volatile solvents and non-volatile components, such as plasticizers [6,7,8]. Hypromellose is widely used as a film-forming polymer due to its favorable film-forming properties and good biocompatibility [9]. Its bioadhesive properties, together with the ability of the formed film to resist friction and maintain close contact with the skin, may contribute to prolonged residence of the formulation at the application site [3,10] and subsequent drug release [11]. Moreover, the combination of volatile and non-volatile solvents can be tailored to achieve an appropriate spreading of FFSs, drying time, appearance, and mechanical characteristics of the resulting films [3,7] that represent one of the most important indicators of FFS performance and integrity after application to the skin [3,12]. Isopropyl alcohol (isopropanol) is frequently incorporated into FFSs as a volatile solvent [13,14,15,16,17]. Its low surface tension (20.93 mN/m at 25 °C), relatively high vapor pressure (4320 Pa at 20 °C), and complete miscibility with water are favorable properties for its use as a solvent component in FFSs, enabling the preparation of homogeneous solutions with relatively short drying times after application to the skin [18]. Its concentration may also affect the formulation’s viscosity and the microstructure and mechanical properties of the resulting film [15,17]. Plasticizers, as non-volatile solvents, are commonly incorporated into FFSs to improve the flexibility of the resulting films, which is particularly important for topical applications where the film must accommodate skin movement and conform to the viscoelastic properties of the skin [7]. By interacting with polymer chains and increasing their mobility, plasticizers can reduce the glass transition temperature of the polymer matrix and facilitate film formation at lower temperatures [8]. Among the plasticizers used in FFSs, liquid macrogols, particularly macrogol 300 and macrogol 400, are frequently employed due to their compatibility with a range of film-forming polymers [17,19,20,21,22,23]. Their plasticizing effect has been demonstrated to improve flexibility and modify the mechanical properties of polymeric films [15,17]; however, these effects are generally concentration-dependent and may only be observed within a specific concentration range. At higher concentrations, excessive plasticizer incorporation may lead to phase separation and reduced plasticizer incorporation into the polymeric matrix, potentially compromising the mechanical properties of the resulting film [17]. Consequently, optimizing the concentrations of solvents in FFS is essential for achieving a suitable balance between drying behavior and the mechanical properties of the resulting film. In our previous study, hypromellose-based FFSs were investigated with respect to the effects of type and concentration of polymer, volatile solvents, and plasticizers on the applicative properties of the FFSs and the resulting films [15]. The study showed that changes in formulation composition caused the greatest variability in the mechanical properties of films [15]. Although this investigation provided a suitable formulation basis, macrogol 400 concentration was considered the only formulation variable, whereas the proportion of the volatile solvent was not taken into account. Clarifying the influence of solvents on applicative properties of FFSs and films is particularly relevant, as they play an important role in solubilizing the drug substance and maintaining the formulation in a suitable liquid state prior to application as well as in the process of its transformation into a film [3]. Therefore, the present study builds upon this previous work with the aim of simultaneously evaluating the effects of macrogol 400 and isopropanol concentrations and their interaction in hypromellose-based FFSs through a Design of Experiments (DoE) approach.

DoE represents a systematic approach to pharmaceutical product and process development that uses predefined objectives and sound statistical principles to obtain maximum information from a minimum number of experiments. In contrast to conventional one-factor-at-a-time approaches, DoE enables systematic investigation of the effects of multiple input factors on selected output responses, including possible interactions between factors [24]. By reducing the number of experiments required, DoE can save considerable time, effort, and resources while improving the understanding of product and process performance and facilitating the development of robust formulations [25]. Depending on the objective, screening designs can be used to identify high-risk variables, whereas optimization designs enable the determination of factor settings that provide the desired response values. Response surface methodology designs are particularly useful for formulation and process optimization, as they provide maximum information with a relatively limited number of experiments and allow the evaluation of both main and interaction effects, thereby supporting the development of mathematical models [26]. Among the experimental designs used for response optimization, factorial designs are widely applied due to their ability to address different experimental objectives. Full factorial designs, in particular, allow factor optimization at two or three levels and, unlike fractional factorial designs, enable the assessment of factor interactions and quadratic effects [27]. Several studies have employed DoE approaches to optimize FFS formulations. Gohel and Nagori [22] and Abdullah and Patil [28] used 3^2^ factorial designs, while Panneerselvam and Somaskanthan applied a 2^3^ full factorial design to optimize FFS formulations [29]. Pawar et al. employed a central composite design [30], whereas Dhimar et al. used a Box–Behnken design within the response surface methodology framework [31]. These studies investigated the effects of different formulation variables, including concentrations of film-forming polymers (Eudragit^®^ [22,28,29,31] and ethyl cellulose [22,29]), plasticizers (sorbitol [28] and propylene glycol [30,31]), volatile solvent (ethanol) [31], and other excipients like pH adjusting agents (sodium citrate [30] and sodium bicarbonate [31]), penetration enhancer (eutectic mixture of camphor and menthol) [29] and active ingredient (luliconazole) [31], on responses such as pH [31], viscosity [28,29], density [29], drug content [30], drying time [28,30,31] and in vitro drug release [22,29,31]. However, to the best of our knowledge, the effects and potential interactions of macrogol 400 and isopropanol concentrations on the mechanical properties of hypromellose-based FFSs have not yet been systematically investigated using a DoE approach.

The present study aimed to develop a hypromellose-based FFS as an advanced carrier for topical administration of ketoprofen using a 3^2^ factorial design to investigate in detail the effects of isopropanol and macrogol 400 concentrations on the mechanical properties of the resulting film, which are essential for maintaining film integrity and accommodating skin movement.

## 2. Materials and Methods

### 2.1. Materials

Hypromellose 2910 (nominal viscosity: 4000 mPa·s, determined for a 2% *w*/*w* aqueous solution at 20 °C; methoxy content 28.0–30.0% and hydroxypropoxy content 7.0–12.0%) (Sigma-Aldrich, St. Louis, MO, USA) was used as the film-forming polymer in placebo and drug-loaded FFSs. Macrogol 400 (polyethylene glycol 400, PEG 400) (Fagron, Rotterdam, The Netherlands) was used as a plasticizer, while isopropanol (isopropyl alcohol) (Fagron, Rotterdam, The Netherlands) and purified water prepared in-house were used as volatile solvents for the preparation of all FFSs. Ketoprofen was purchased from Sigma-Aldrich (St. Louis, MO, USA). All FFS ingredients were of Ph. Eur. quality. Sodium hydroxide and potassium dihydrogen phosphate (Sigma-Aldrich, St. Louis, MO, USA), of analytical grade purity, were used for the preparation of the phosphate buffer.

### 2.2. Experimental Design

The DoE was established using Design-Expert^®^ software (Version 7.0, Stat-Ease Inc., Minneapolis, MN, USA) to evaluate the influence of selected formulation variables on the mechanical properties of the films and to establish the relationship between formulation factors and responses. A 3^2^ full factorial design was applied, with macrogol 400 concentration (X_1_) and isopropanol concentration (X_2_) selected as the independent variables. Each independent variable was investigated at three levels (low, medium, and high), as presented in Table 1. The factor levels were defined based on preliminary studies performed during the formulation development process [15]. Nine placebo FFS formulations (Table 2) were used as the basis for defining the design space. Hypromellose concentration was fixed at 2% for all nine FFSs, although it is an important formulation variable affecting the properties of the FFSs and the resulting films, because its influence had already been systematically investigated in our previous study, where different hypromellose types and concentrations were comprehensively evaluated [15]. In these FFSs, the water/isopropanol mass ratio was set at 3:1, 2:1, or 1:1. For each water/isopropanol mass ratio, FFSs containing 0%, 2.5%, or 5% macrogol 400 were prepared. The mechanical properties of the corresponding films were selected as the dependent or response variables. The response variables selected for the experimental design were tensile strength (Y_1_), elongation at break (Y_2_), and Young’s modulus (Y_3_), which were determined by tensile testing of films prepared from the corresponding nine FFSs. The DoE analysis included data collected in the previous study for FFSs prepared with a water/isopropanol mixture at ratios of 3:1 and 1:1 [15] and those obtained for three additional FFSs comprising a water/isopropanol mixture at a ratio of 2:1, newly prepared and characterized in the present study to complete the 3^2^ full factorial design. The preparation of the three additional FFS formulations with the corresponding films and the evaluation of their mechanical properties, including tensile strength, elongation at break, and Young’s modulus, were performed using the same methodology as that applied to the other six FFS formulations [15]. All nine formulations were prepared and evaluated under identical experimental conditions, following the same procedures for FFS preparation, film casting, and mechanical testing, using the same batches of the respective materials. The obtained mechanical characteristics were used for statistical analysis and development of regression models in Design-Expert^®^ software. The experimental runs and measured response values are presented in Table 2. The data were initially fitted to a quadratic polynomial model, and model reduction was performed by eliminating non-significant terms to obtain the most appropriate reduced model for each response. Analysis of variance (ANOVA) was used to evaluate the significance of the models and individual model terms at a significance level of *p* < 0.05. Model adequacy was evaluated using the coefficient of determination (R^2^), adjusted R^2^, predicted R^2^, coefficient of variation (CV), adequate precision, and residual diagnostics. Predicted-versus-observed plots, normal probability plots of residuals, and residuals-versus-predicted plots were evaluated for each response. Box–Cox analysis was used to assess the need for response transformation. Based on this analysis, Young’s modulus was square-root transformed prior to final model fitting. Also, two-dimensional contour plots and model graphs were generated by the Design-Expert^®^ software.

Based on the developed regression models, numerical optimization was performed using Design-Expert^®^ software to identify the optimal values of the independent variables that simultaneously satisfied the predefined criteria for the investigated responses. The optimization criteria were defined to maximize tensile strength (Y_1_), achieve elongation at break values within the range of 50–120% (Y_2_), and minimize Young’s modulus (Y_3_). These criteria were selected based on the desired mechanical characteristics of topical FFSs, where sufficient tensile strength is required to maintain film integrity, the selected elongation range ensures adequate flexibility, and low Young’s modulus indicates reduced stiffness of the formed films [32]. Numerical optimization using the desirability function was applied to identify the factor settings providing the most favorable combination of the predefined mechanical-response criteria. Numerical optimization was used as a model-guided decision-support step, whereas final formulation selection additionally considered experimentally determined physicochemical, rheological, textural, and film mechanical characteristics of the subsequently prepared formulations.

The relative prediction error (RPE) was calculated as the absolute difference between the observed and predicted values relative to the observed value, according to Equation (1):

RPE (%) = |Observed − Predicted|/Observed ∙ 100.(1)

### 2.3. Preparation of DoE-Based Placebo FFSs

Based on the DoE numerical optimization results, six new placebo FFSs (Table 3) were subsequently prepared and characterized together with the corresponding films.

According to the previously reported procedure [15], a 7.5% (*w*/*w*) hypromellose stock solution was prepared by the hot/cold method, in which hypromellose was dispersed in purified water heated to approximately 90 °C under continuous stirring, followed by cooling below 5 °C for 24 h to allow complete polymer hydration. The required amount of the stock solution was then mixed with the remaining formulation components at 300 rpm using an AREX Digital PRO Heating Magnetic Stirrer (VELP Scientifica, Italy) at room temperature until homogeneous formulations were obtained.

### 2.4. Characterization of DoE-Based Placebo FFSs

To characterize DoE-based placebo FFSs, their visual appearance, pH, and transparency were evaluated. Rheological and textural analyses were additionally performed to assess the flow behavior and consistency of the FFSs, as these properties may influence their handling and spreading during application, as well as their ability to remain at the site of application during metamorphosis into film [33,34,35].

#### 2.4.1. Assessment of Visual Appearance

The visual appearance of FFSs was evaluated by visual inspection under ambient light conditions against a white and black background to assess homogeneity, transparency, and the presence of visible particles or phase separation.

The transparency of the FFSs was evaluated by determining their %T using an Evolution 300 UV–Vis spectrophotometer (Thermo Scientific, Waltham, MA, USA) at a wavelength of 600 nm. %T reflects the amount of light transmitted through the sample and was used as an indirect indicator of solution homogeneity and the mutual solubility of components [36]. Based on previous reports, formulations with %T values higher than 90% were considered to exhibit complete dissolution of the formulation components [36].

#### 2.4.2. pH Measurement

The pH of the FFSs was determined at room temperature (20 ± 3 °C) using a previously calibrated (standard buffer solutions of pH 4.0 and 7.0) digital pH meter (HI 2223, HANNA Instruments, Woonsocket, RI, USA). An aliquot of each FFS was placed in a laboratory beaker, and the pH electrode was immersed in the sample. The pH value was recorded after the reading had stabilized for 5 min.

#### 2.4.3. Rheological Characterization of FFSs

The rheological behavior of the placebo FFSs was evaluated using a RheolabMC120 rotational rheometer (Anton Paar, Ostfildern, Germany) equipped with an MK22 cone-and-plate measuring system (50 mm cone diameter, 50 µm gap, and 1° cone angle). The FFS sample was carefully loaded onto the measuring plate, and the cone was immediately lowered to the predefined measuring gap to minimize isopropanol evaporation and prevent potential changes in the FFS composition and, consequently, its viscosity and rheological structure. Flow measurements were performed in triplicate by applying a controlled shear rate from 0 to 200 s^−1^, followed by a decreasing shear rate from 200 to 0 s^−1^. Prior to each measurement, the samples were allowed to equilibrate for 30 s under zero-shear conditions (0 s^−1^). All measurements were conducted at 20 ± 0.1 °C, representing typical ambient storage conditions [37], and at 32 ± 0.1 °C, corresponding to the temperature of the skin surface [14].

The rheological behavior of each formulation was characterized based on the obtained flow curves. The ascending flow curve was fitted to the Ostwald–de Waele (power-law) model to determine the consistency index (*k*) and flow behavior index (*n*), according to Equation (2) [34]:

*σ* = *k* ∙ *γ^n^*, (2)

where *σ* is the shear stress (Pa), *k* is the consistency index [(Pa·s)^n^], *γ* is the shear rate (s^−1^), and *n* is the flow behavior index (dimensionless).

The apparent viscosity was determined at shear rates of 10.5 s^−1^ and 200 s^−1^, corresponding to the maximum (*η*_max_) and minimum (*η*_min_) apparent viscosity, respectively. The hysteresis area (HA) was calculated using US200 software (Anton Paar, Ostfildern, Germany). All rheological parameters (*k*, *n*, *η*_max_, *η*_min_ and HA) are presented as mean ± standard deviation of three independent measurements.

#### 2.4.4. Texture Profile Analysis of Placebo FFSs

Texture analysis of the FFSs was performed using an EZ-LX texture analyzer (Shimadzu, Kyoto, Japan) using a compression test. A 22.0 g sample of each FFS was transferred into glass beakers (34 mm in diameter and 50 mm in height). The measurement procedure involved lowering an aluminum cylindrical probe (40 mm in length and 20 mm in diameter) toward the sample surface at a constant speed of 2 mm/s, with a pre-test force of 0.02 N. Upon contact with the sample surface, the probe continued to penetrate the sample to a depth of 20 mm at the same speed. Subsequently, the probe was withdrawn vertically over a distance of 30 mm at a speed of 2 mm/s. Force–distance profiles were recorded using TrapeziumX software (version 1.5.2, Shimadzu, Kyoto, Japan), and textural parameters were calculated from the generated force–distance curves. The evaluated parameters included firmness (N), consistency (mJ), adhesiveness (mJ), cohesiveness (N), and stringiness (mm). All measurements were performed in triplicate.

Firmness was defined as the maximum force required for probe penetration into the sample and corresponded to the peak positive force observed during the compression phase of the force–distance curve, reflecting the resistance of the formulation to deformation. Consistency was calculated as the positive area under the force–distance curve during compression and expressed as work (mJ), representing the energy required to deform the sample. Adhesiveness was calculated as the negative area under the force–distance curve during the withdrawal phase and represented the work required to overcome adhesive forces between the sample and the probe. Cohesiveness was determined as the maximum negative force recorded during probe withdrawal and reflected the ability of the formulation to resist separation from the probe. Stringiness was expressed as the maximum distance traveled by the probe while maintaining contact with the sample before rupture of the formed filament. Parameters obtained from the negative portion of the curve were expressed as absolute values.

### 2.5. Preparation of Films from DoE-Based Placebo FFSs

Films were prepared by the solvent casting method according to the previously reported methodology [15]. Briefly, 6.0 g of each FFS was poured into the bottom of a glass Petri dish (9 cm in diameter) to obtain a uniform layer. The samples were allowed to dry uncovered under controlled conditions (21 ± 1 °C and 40 ± 5% relative humidity) for 48 h. The resulting films were carefully peeled from the Petri dishes and stored in hermetically sealed polyethylene bags until further characterization. For each formulation condition, one FFS batch was prepared, from which three films were independently cast under identical controlled conditions and used as replicates for mechanical characterization.

### 2.6. Characterization of Films Prepared from DoE-Based Placebo FFSs

The films obtained from the placebo FFSs were characterized in terms of their mechanical properties. In addition, visual appearance, weight, and stickiness were assessed as quality aspects of the formed films important for patient acceptability.

#### 2.6.1. Assessment of Visual Appearance of Films

The visual appearance of films is an important factor influencing patient acceptability, as transparent films may be more cosmetically acceptable for application to the skin. The prepared films were visually examined for their transparency and surface uniformity, as well as for the presence of air bubbles. In addition to their potential impact on the visual appearance of the films, a non-uniform surface and air bubbles may compromise structural uniformity and consequently affect their mechanical properties.

#### 2.6.2. Determination of Film Weight

The weight of each film was determined using a high-precision digital balance (Precision Balance PS 750.R2, RADWAG Wagi Elektroniczne, Radom, Poland; readability 0.001 g). Film weight was calculated by subtracting the weight of the empty Petri dish from the weight of the Petri dish containing the dried film. The results are presented as mean ± standard deviation.

#### 2.6.3. Determination of Film Thickness

The thickness of each film specimen was measured at five different locations, including the center and four corners, using a digital micrometer (Digital Micrometer MCMD025, KG KERN, Balingen, Germany). The results are presented as mean ± standard deviation.

#### 2.6.4. Assessment of Film Stickiness

Film stickiness was assessed according to the previously reported methodology by Milinković et al. [15]. Briefly, a piece of cotton wool was allowed to fall freely onto the surface of the dry film. The bottom of a Petri dish was then placed over the cotton wool and kept in contact with the film surface for 1 min. After removal of the Petri dish and cotton wool, the presence of cotton fibers adhering to the film surface was visually assessed. Stickiness was classified as low (occasional or no fiber adhesion), moderate (a thin layer of fibers observed on the film surface), or high (a dense layer of cotton fibers remaining adhered to the film) [15,38].

#### 2.6.5. Determination of Mechanical Properties of Films

The mechanical properties of the films were evaluated using an EZ-LX texture analyzer (Shimadzu, Kyoto, Japan) equipped with a 1000 N load cell. Data acquisition and instrument control were performed using TrapeziumX software (version 1.5.2, Shimadzu, Kyoto, Japan). The tensile testing procedure was performed in accordance with the international standard ISO 527-3 (Plastics—Determination of tensile properties, Part 3: Test conditions for films and sheets) [39]. Prior to testing, the films were cut into dumbbell-shaped specimens with an overall length of 76 mm and a width of 13 mm in the narrow section [39]. The thickness of each specimen was measured at five different locations, including the center and four corners, using a digital micrometer (Digital Micrometer MCMD025, KG KERN, Balingen, Germany), and the mean value was used for subsequent calculations. The specimens were vertically mounted between two pneumatic grips with an initial grip separation of 50 mm, corresponding to a gauge length of 50 mm. The upper grip was then moved upward at a constant crosshead speed of 1 mm/s until specimen rupture occurred [40]. All measurements were determined under controlled environmental conditions at a temperature of 21 ± 1 °C and relative humidity of 40 ± 5%. The primary parameters obtained from the force–displacement curves were the maximum force and the initial slope of the curve. The maximum force represents the highest force sustained by the film before rupture, whereas the initial slope reflects the force required to produce a given deformation in the initial, approximately linear region of the force–displacement curve. These parameters were subsequently used to calculate the tensile strength (TS), elongation at break (EB), and Young’s modulus (YM). Tensile strength was calculated using Equation (3):

TS = *F*_m_/(*L* ∙ *W*), (3)

where *F*_m_ represents the maximum force required to break the film (N), *L* is the average film thickness (mm), and *W* is the initial width of the specimen (mm) [15].

Elongation at break was determined according to Equation (4):

EB = (*L*_max_ − *L*_0_)/*L*_0_ ∙ 100%, (4)

where *L*_max_ is the maximum length of the specimen immediately before rupture (mm), while *L*_0_ corresponds to its initial length (mm) [15].

Young’s modulus was obtained from the slope of the initial linear region of the stress–strain curve [15]. All measurements were performed in triplicate, and the results are presented as mean ± standard deviation. The reported mean ± SD values therefore represent variability among independently cast films obtained from the same FFS batch.

### 2.7. Preparation and Characterization of Ketoprofen-Loaded FFS and Corresponding Film

Following the characterization of the six DoE-based placebo FFSs prepared after numerical optimization and their corresponding films, the formulation showing the most suitable overall characteristics was selected as the vehicle for preparation of a ketoprofen-loaded FFS containing 2.5% (*w*/*w*) ketoprofen. Ketoprofen was added to the suitable amount of selected placebo FFS under continuous stirring (300 rpm) using a magnetic stirrer (AREX Digital PRO Heating Magnetic Stirrer, VELP Scientifica, Usmate, Italy), at room temperature, until complete dissolution was achieved.

The ketoprofen-loaded FFS was also evaluated with respect to pH, transparency, rheological and textural properties. All analyses were conducted using the same procedures as those previously applied to the placebo FFSs. Additionally, in vitro drug release from the ketoprofen-loaded FFS was evaluated. The ketoprofen-loaded FFSs were used to prepare films according to the procedure described in Section 2.5. The obtained films, following 48 h of drying, were also assessed for their visual appearance, weight, stickiness, and mechanical properties, including tensile strength, elongation at break, and Young’s modulus, using the same procedures as those described for the placebo films.

### 2.8. In Vitro Ketoprofen Release Testing

The in vitro release of ketoprofen from the ketoprofen-loaded FFS was evaluated using a USP II dissolution apparatus with a rotating paddle (Erweka DT126 Light, Erweka, Langen, Germany) equipped with an immersion cell (VanKel Technology Group Inc., Cary, NC, USA). A precisely weighed amount of 2.0 g of formulation was placed in the immersion cell and covered with synthetic nitrocellulose Porafil^®^ membrane filters (0.45 µm pore size; Macherey-Nagel, Düren, Germany). The release study was performed at 32 ± 1 °C using 500 mL of phosphate buffer (pH 7.2) as the acceptor medium, with a paddle rotation speed of 50 rpm. Samples of the acceptor medium were withdrawn at predetermined time intervals (0.25, 0.5, 1, 1.5, 2, 3, 4, 5, and 6 h), and the withdrawn volume was immediately replaced with an equal amount of fresh buffer solution. The concentration of ketoprofen in the collected samples was determined spectrophotometrically using an Evolution 300 UV–Vis spectrophotometer (Thermo Scientific, Waltham, MA, USA) at the wavelength of maximum absorption of ketoprofen (260 nm). The release study was performed in triplicate, and the results were expressed as the mean cumulative amount of released ketoprofen ± standard deviation. The ketoprofen release profile was analyzed using a model-dependent approach in Microsoft Excel (Version 16.101.3 (25100321), Microsoft, Redmond, WA, USA). The experimental data were fitted to first-order, Higuchi, Korsmeyer–Peppas, and Weibull kinetic models. The Akaike Information Criterion (AIC) was calculated for each model according to the approach described by Azadi et al. [41] to evaluate the goodness of fit and select the most appropriate kinetic model describing ketoprofen release from the tested formulation.

### 2.9. Statistical Analysis

ANOVA was used to evaluate the significance of the factorial design models and individual model terms at a significance level of *p* < 0.05 using Design-Expert^®^ software (Version 7.0, Stat-Ease Inc., Minneapolis, MN, USA). As independent FFS-batch replication at identical factor settings was not included in the factorial design, batch-level pure error and formal lack-of-fit were not estimated. Additionally, statistical analysis was performed on the results of textural measurements of FFSs and on the mechanical properties of films obtained from FFSs. Due to the limited sample size, formal assessment of data normality was not performed. Consequently, parametric ANOVA was applied consistently to evaluate the effects of macrogol 400 concentration and isopropanol content on the investigated responses. Statistical analyses were performed using R software (R Statistical Software version 4.4.1, R Core Team 2024, The R Foundation for Statistical Computing, Vienna, Austria) [42] and the RStudio programming environment (RStudio: Integrated Development Environment for R version 2025.5.0.496, Posit Software, PBC, Boston, MA, USA) [43]. All differences with *p* < 0.05 were considered statistically significant.

## 3. Results

### 3.1. Factorial Design

A 3^2^ full factorial design was applied to nine placebo FFSs to evaluate the influence of the selected formulation variables on the mechanical properties of the resulting films and to optimize their levels in the final FFS formulation. The independent variables included macrogol 400 concentration (X_1_) and isopropanol concentration (X_2_). Based on preliminary experiments, macrogol 400 was investigated at three levels: low (0%), medium (2.5%) and high (5%, *w*/*w*). The isopropanol concentration was also investigated at three levels: 25%, 33% and 50% (*w*/*w*), corresponding to the low, medium and high levels, respectively. The experimentally obtained values of the response variables ranged from 7.17 to 65.74 MPa for tensile strength (Y_1_), from 3.26 to 123.52% for elongation at break (Y_2_), and from 34.66 to 3297.59 MPa for Young’s modulus (Y_3_) (Table 2), indicating that changes in formulation composition markedly affected the mechanical properties of the films.

Mathematical models describing the relationship between the concentrations of the solvents as formulation variables and the investigated responses were generated by fitting the experimental data to polynomial equations using multiple regression analysis. The significance of the investigated factors was assessed by ANOVA. A significance threshold of 5% was adopted, with *p*-values below 0.05 indicating a statistically significant effect. The results showed that isopropanol concentration (X_2_) did not significantly affect tensile strength, elongation at break, or Young’s modulus (*p* > 0.05). In contrast, macrogol 400 concentration (X_1_) significantly affected all three investigated responses (*p* < 0.05).

Accordingly, numerical optimization was performed by varying the macrogol 400 concentration, while isopropanol content was excluded from the final reduced models as it did not significantly influence the principal mechanical responses. The optimization criteria included maximizing tensile strength, targeting an elongation at break of 50–120%, and minimizing Young’s modulus. Numerical optimization of the final response models yielded a maximum overall desirability of approximately 0.474 at a macrogol 400 concentration of approximately 2.56% (*w*/*w*). Since isopropanol concentration was not retained in the final mechanical-response models, it did not affect numerical desirability within the investigated range. Based on this model-guided result, formulations containing 2.5%, 2.75%, and 3.0% (*w*/*w*) macrogol 400 were subsequently investigated experimentally at two water/isopropanol ratios to refine formulation selection using a broader set of performance attributes. Thus, six DoE-based placebo FFSs (Table 3) containing 2.5%, 2.75%, or 3.0% (*w*/*w*) macrogol 400 and water/isopropanol ratios of 2:1 or 1:1 were prepared and subsequently characterized. The experimentally determined mechanical properties of the formulation that most closely matched the predicted responses were compared with the corresponding predicted values, as presented in Table 4.

Observed values are presented as mean ± SD. Predicted values represent model-based point predictions. The 95% prediction interval represents the expected range for a future experimental response at the same factor settings. For Young’s modulus, the predicted value and prediction interval were obtained from the square-root-transformed model and back-transformed to the original scale. Relative prediction error was calculated as |observed − predicted|/observed × 100. The overall desirability calculated for the experimentally selected formulation was approximately 0.395, whereas the maximum model-based desirability within the investigated domain was approximately 0.474.

#### Effect of Macrogol 400 Concentration on Mechanical Properties of Films

The quadratic model fitted to the tensile strength (Y_1_) data of the films formed from placebo FFSs was statistically significant (F = 186.16, *p* < 0.0001) and is given by Equation (5):

Y_1_ = 18.39 − 25.51X_1_ + 19.04X_1_^2^, (5)

where Y_1_ represents tensile strength, and X_1_ represents the coded value of macrogol 400 concentration. Among the evaluated model terms, both the linear (X_1_) (F = 314.03, *p* < 0.0001) and quadratic (X_1_^2^) (F = 58.29, *p* = 0.0003) terms of macrogol 400 concentration were statistically significant.

**Figure 1:**
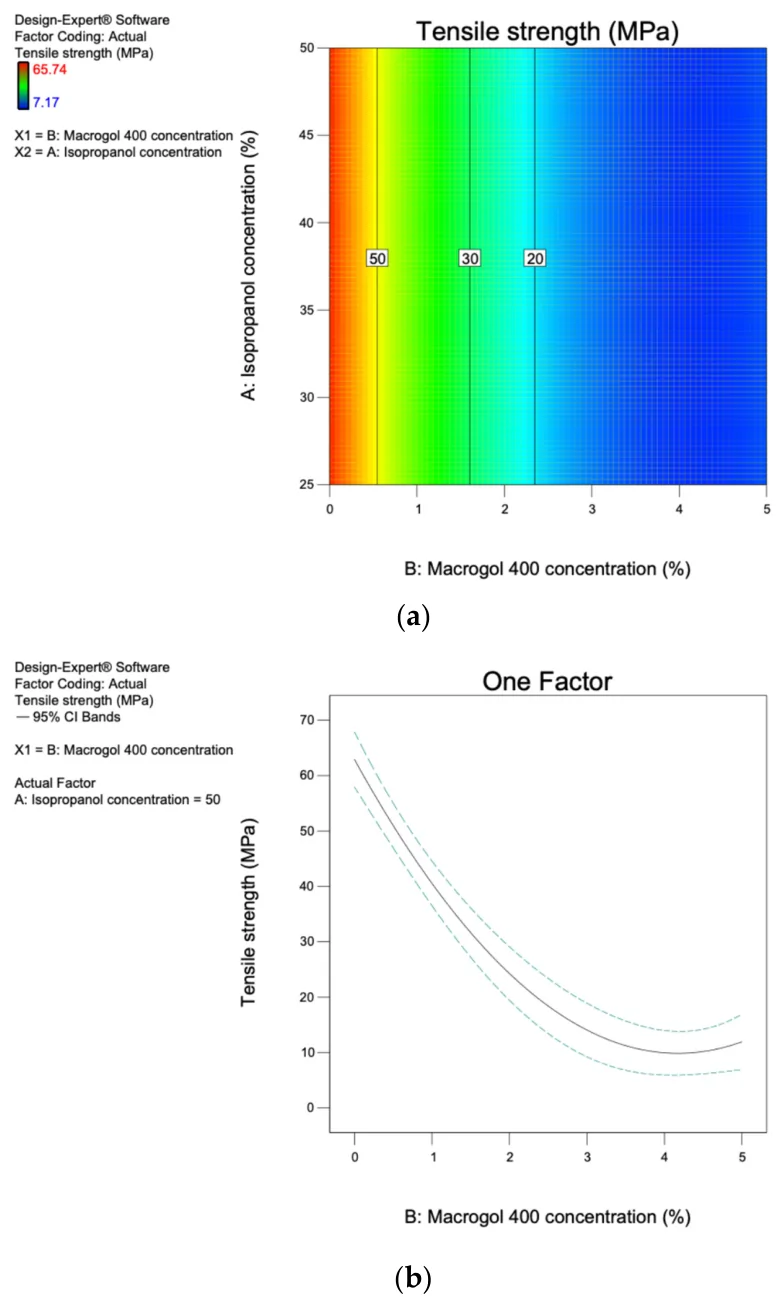
Contour plot (**a**) and model plot (**b**) illustrating the effect of macrogol 400 concentration (X_1_) on the tensile strength (Y_1_) of films formed from placebo FFSs. Dashed lines indicate the 95% confidence interval of the fitted model.

The negative linear coefficient indicates an overall decrease in tensile strength with increasing macrogol 400 concentration, whereas the positive quadratic coefficient indicates that the relationship is nonlinear across the investigated concentration range. Consequently, the model predicts a minimum in tensile strength, followed by an increase as the macrogol 400 concentration is further increased (Figure 1).

**Figure 2:**
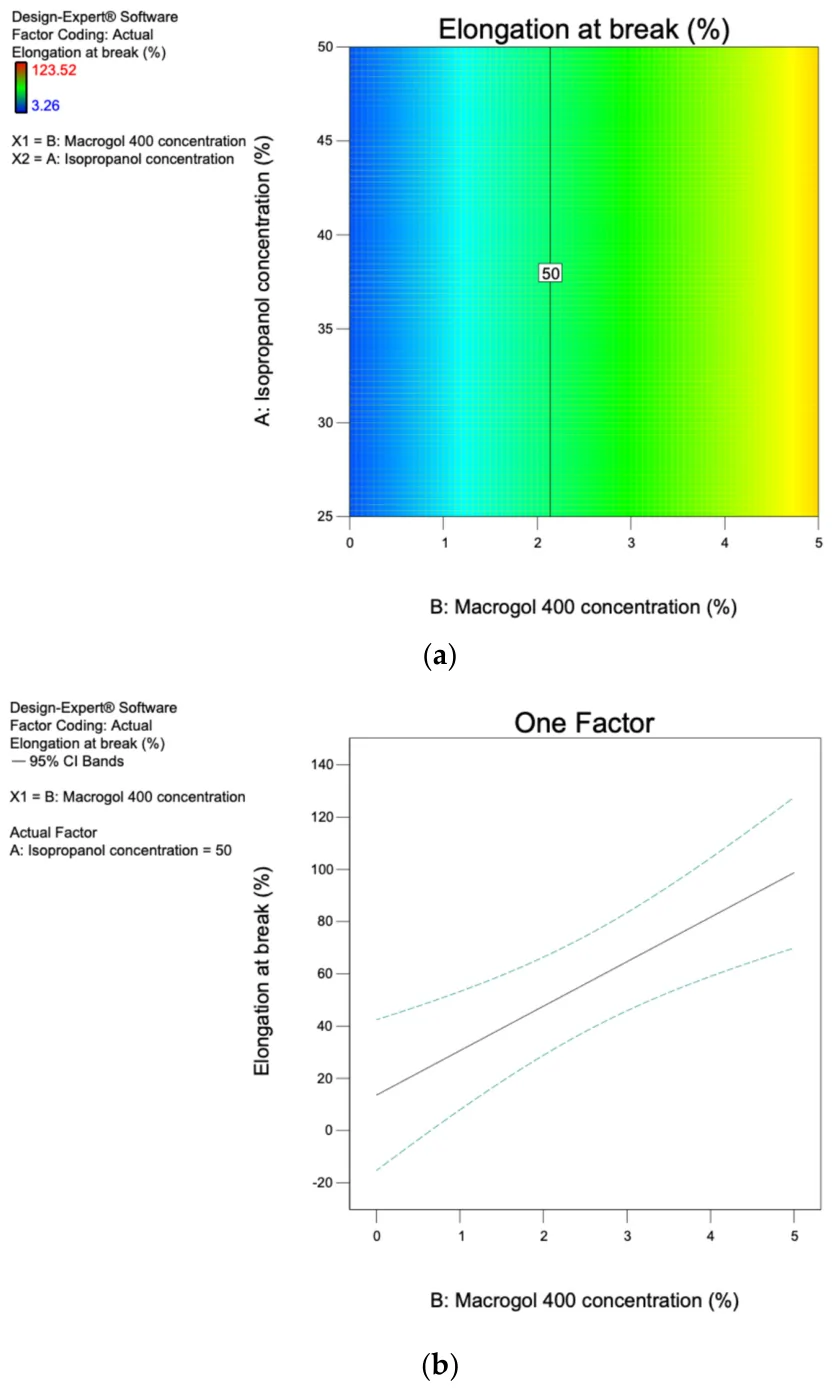
Contour plot (**a**) and model plot (**b**) illustrating the effect of macrogol 400 concentration (X_1_) on the elongation at break (Y_2_) of films formed from placebo FFSs. Dashed lines indicate the 95% confidence interval of the fitted model.

The elongation at break of the films was adequately described by a statistically significant linear model (F = 20.30, *p* = 0.0028), with macrogol 400 concentration (X_1_) identified as the only significant model term (F = 20.30, *p* = 0.0028). The resulting regression equation is presented in Equation (6):

Y_2_ = 56.15 + 42.59X_1_, (6)

where Y_2_ represents elongation at break and X_1_ represents the coded macrogol 400 concentration. The positive coefficient of the linear term indicates that increasing macrogol 400 concentration resulted in a progressive increase in elongation at break (Figure 2). This linear relationship is also reflected in the model plot (Figure 2b), which shows a consistent increase in the predicted elongation at break with an increase in macrogol 400 concentration across the investigated macrogol 400 concentration range.

**Figure 3:**
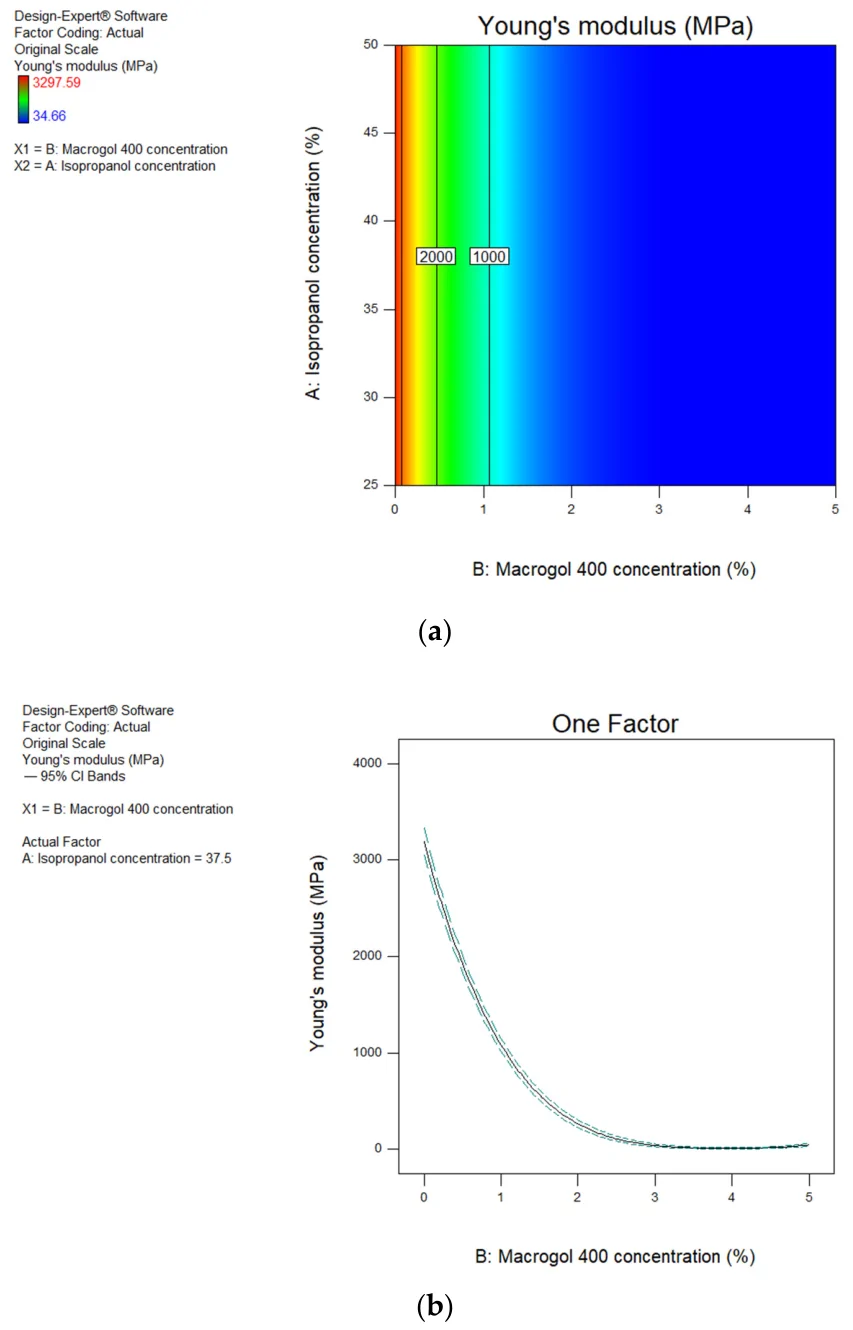
Contour plot (**a**) and model plot (**b**) illustrating the effect of macrogol 400 concentration (X_1_) on the Young’s modulus (Y_3_) of films formed from placebo FFSs. Dashed lines indicate the 95% confidence interval of the fitted model.

Box–Cox analysis indicated that a transformation was appropriate for Young’s modulus, with an estimated λ of 0.36 and a 95% confidence interval of 0.03–0.65. Based on the Box–Cox analysis, a square-root transformation (λ = 0.5) was selected for model fitting. The square-root-transformed Young’s modulus data were adequately described by a statistically significant quadratic model (F = 2922.65, *p* < 0.0001), with both the linear (F = 4698.01, *p* < 0.0001) and quadratic terms (F = 1147.29, *p* < 0.0001) of macrogol 400 concentration contributing significantly to the response. The resulting regression equation is presented in Equation (7):

√Y_3_ = 10.37 − 24.86X_1_ + 21.28X_1_^2^, (7)

where Y_3_ represents Young’s modulus, and X_1_ represents the coded value of macrogol 400 concentration. The negative linear coefficient indicates an overall decrease in Young’s modulus with increasing macrogol 400 concentration, whereas the positive quadratic coefficient indicates that the relationship does not remain linear across the investigated concentration range (Figure 3). Thus, the model predicts a minimum in Young’s modulus, followed by an increase at higher macrogol 400 concentrations.

Model adequacy was further assessed using model-fit and predictive statistics. For tensile strength, R^2^, adjusted R^2^ and predicted R^2^ were 0.9841, 0.9789 and 0.9643, respectively, with a CV of 11.34% and adequate precision of 25.061. For elongation at break, the corresponding values were 0.7436, 0.7069, and 0.5507, with a CV of 41.25% and adequate precision of 7.803, indicating greater variability and lower predictive performance for this response. Box–Cox analysis indicated that square-root transformation was appropriate for Young’s modulus. The resulting reduced quadratic model showed R^2^ = 0.9990, adjusted R^2^ = 0.9986, predicted R^2^ = 0.9977, CV = 3.62%, and adequate precision = 96.933. Complete model statistics and diagnostic plots are provided in the Supplementary Material.

### 3.2. Characteristics of DoE-Based Placebo FFSs

All placebo FFSs appeared as clear, homogeneous, and colorless liquids upon visual inspection, with no visible particles or signs of phase separation. The pH values of placebo FFSs ranged from 4.98 to 5.62 (Table 5). Formulations with a water/isopropanol mixture 1:1 (WI 11 group) exhibited slightly higher pH values (5.57–5.62) than those with a 2:1 water/isopropanol mixture (WI 21 group) (4.98–5.01). Within each solvent ratio, variations in macrogol 400 concentration did not affect the pH values of the placebo FFSs.

All placebo FFSs exhibited high %T values (>97%), with only minor differences observed between formulations (Table 5).

**Figure 4:**
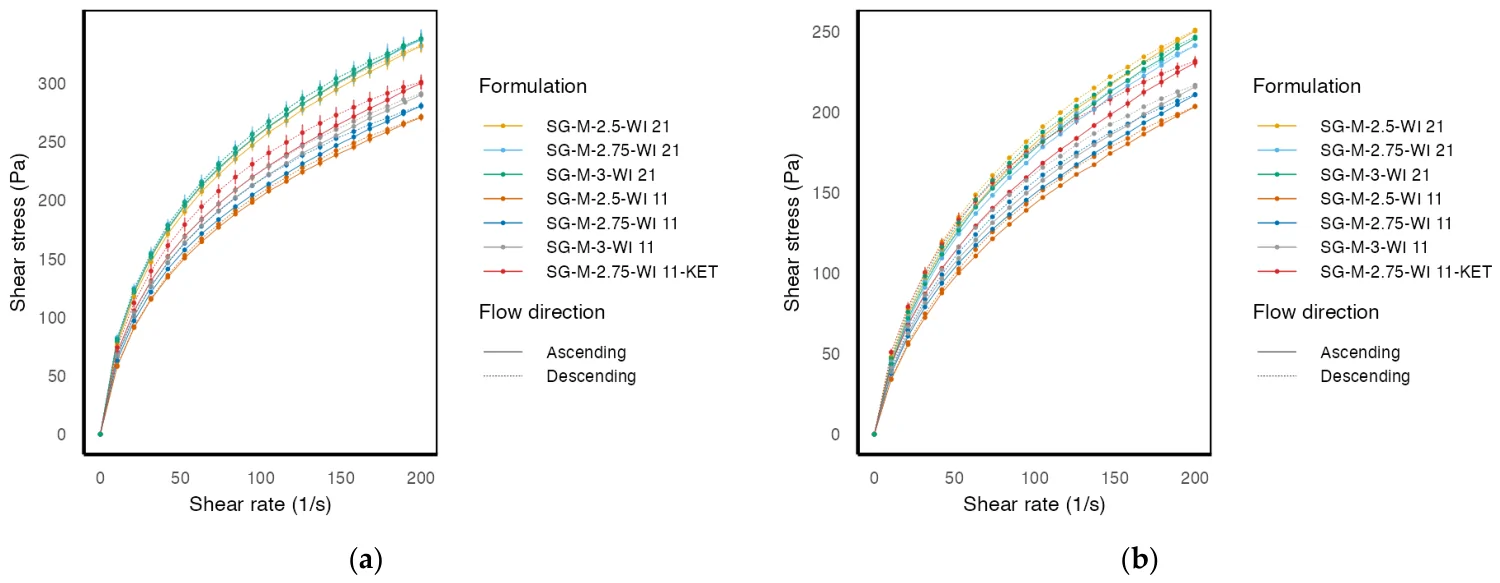
Flow curves of DoE-based placebo FFSs and ketoprofen-loaded FFS (designated with the suffix “-KET”) at (**a**) 20 °C and (**b**) 32 °C.

The flow behavior of the investigated placebo FFSs at 20 °C was evaluated based on the flow curves obtained by plotting shear stress as a function of shear rate (Figure 4a). All placebo FFSs exhibited a non-linear increase in shear stress with increasing shear rate, with a progressive reduction in the slope of the curves at higher shear rates, indicating shear-thinning behavior. This observation was further confirmed by the *n* values below 1 (Table 6). In addition, hysteresis loops were observed between the ascending and descending flow curves. The corresponding HA values, together with the remaining rheological parameters, are presented in Table 6.

At 20 °C, both the *η*_max_ and *η*_min_ values were higher for FFSs in the WI 21 group than for those in the WI 11 group (Table 6). The effect of macrogol 400 concentration depended on the isopropanol content. Within the WI 11 group, both the *η*_max_ and *η*_min_ increased significantly with increasing macrogol 400 concentration up to 2.75%, whereas a further increase to 3% did not produce a statistically significant difference. In the WI 21 group, a statistically significant difference in apparent viscosity was observed only between FFSs containing 2.5% and 2.75% macrogol 400. The *k* values followed the same trends.

At 32 °C, all placebo FFSs also exhibited pseudoplastic shear-thinning behavior (Figure 4b). Both the *η*_max_ and *η*_min_ values were lower than those measured at 20 °C (Table 6). Nevertheless, the same trends associated with isopropanol content and macrogol 400 concentration were maintained. The *k* values were also lower at 32 °C, whereas the *n* and HA values were higher (Table 6). The differences between 20 °C and 32 °C were statistically significant for all evaluated rheological parameters.

**Figure 5:**
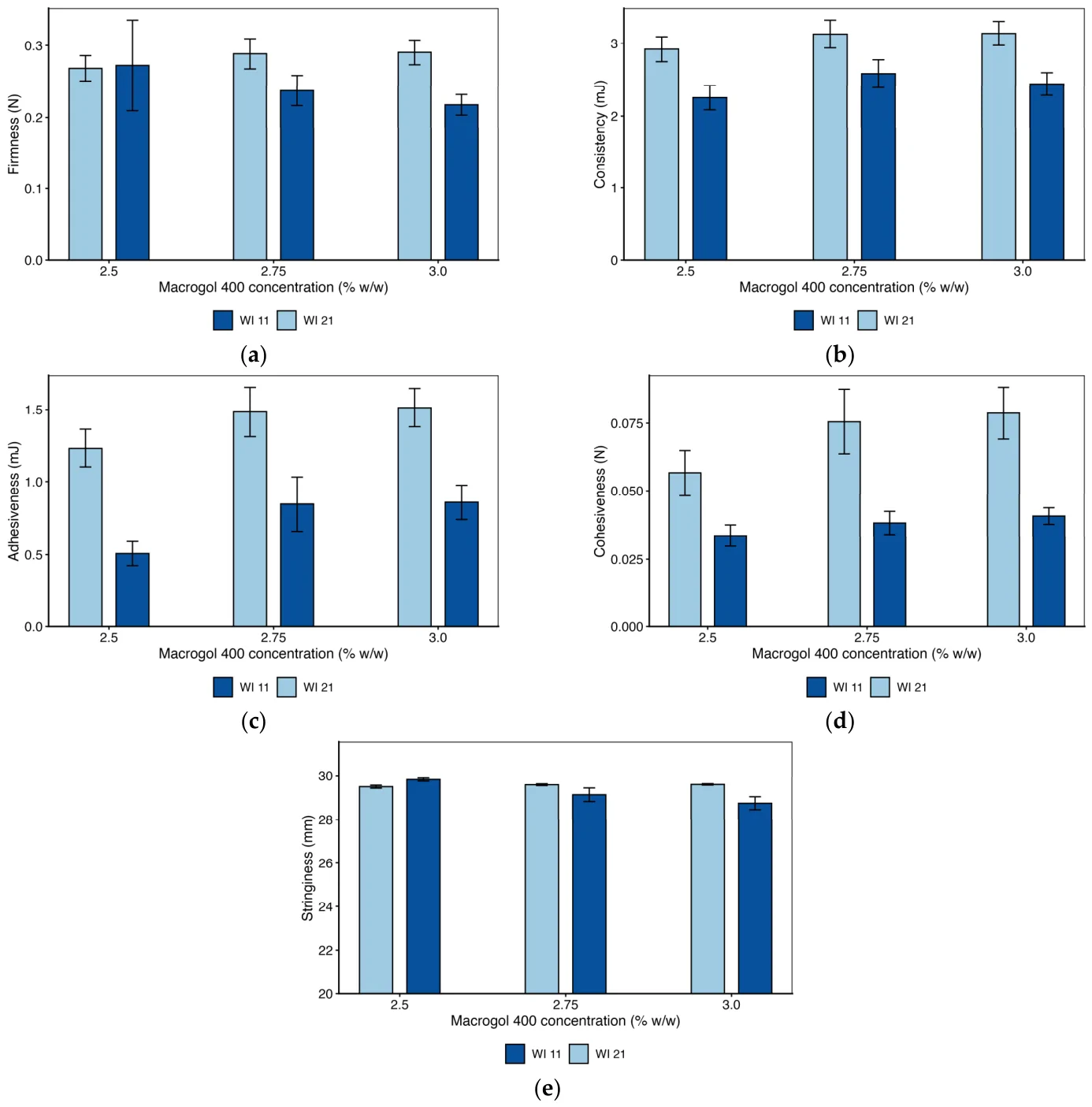
Effect of macrogol 400 concentration and isopropanol content on the texture profile analysis parameters: (**a**) firmness, (**b**) consistency, (**c**) adhesiveness, (**d**) cohesiveness, and (**e**) stringiness of DoE-based placebo FFSs.

The textural characteristics of the DoE-based placebo FFSs are presented in Table 7. All FFSs exhibited relatively low firmness values, ranging from 0.2177 to 0.2904 N. A clear difference was observed between the two FFS groups, as WI 21 group FFSs exhibited higher firmness than WI 11 group FFSs (a difference of 0.040 (95% CI: 0.008, 0.071), *p* = 0.0173, n = 18, Cohen’s d = 1.30 (95% CI for Cohen’s d: 0.122, 2.48)) (Table 7, Figure 5a). The effect of macrogol 400 concentration differed according to the FFS group. In WI 21 group FFSs, increasing macrogol 400 concentration from 2.5% to 3% resulted in a slight increase in firmness, with values increasing from 0.2684 ± 0.0175 N to 0.2904 ± 0.0171 N. In contrast, WI 11 group FFSs exhibited a gradual decrease in firmness with increasing macrogol 400 concentration, from 0.2724 ± 0.0629 N to 0.2177 ± 0.0144 N.

Consistency values were also higher for WI 21 group FFSs (2.9200–3.1378 mJ) than for the corresponding WI 11 group FFSs (2.2551–2.5858 mJ) (a difference of 0.634 (95% CI: 0.455, 0.812), *p* = 5.26 × 10^−6^, n = 18, Cohen’s d = 3.65 (95% CI for Cohen’s d: 1.73, 5.57)) (Table 7, Figure 5b). Also, a statistically significant difference was observed between formulations containing 2.5% and 2.75% macrogol 400 (a difference of 0.270 (95% CI: 0.002, 0.537), *p* = 0.0482, n = 18, Cohen’s d = 1.553 (95% CI for Cohen’s d: 0.118, 2.988)), whereas no significant difference was observed between the other macrogol 400 concentrations.

The differences between the two FFS groups were also reflected in adhesiveness and cohesiveness (Table 7, Figure 5c,d). Both the adhesiveness (a difference of 0.672 (95% CI: 0.528, 0.816), *p* = 3.03 × 10^−7^, n = 18, Cohen’s d = 4.79 (95% CI for Cohen’s d: 2.42, 7.15)) and cohesiveness (a difference of 0.033 (95% CI: 0.025, 0.040), *p* = 9.38 × 10^−7^, n = 18, Cohen’s d = 4.31 (95% CI for Cohen’s d: 2.13, 6.48)) were higher in WI 21 group FFSs than in WI 11 group FFSs. In WI 21 group FFSs, increasing the macrogol 400 concentration from 2.5% to 3% was accompanied by an increase in adhesiveness from 1.2324 ± 0.1317 mJ to 1.5130 ± 0.1321 mJ and in cohesiveness from 0.0566 ± 0.0084 N to 0.0787 ± 0.0095 N. The macrogol 400 concentration-dependent effects observed for adhesiveness and cohesiveness were statistically significant (*p* = 0.00339 and *p* = 0.0136, respectively, n = 18). As observed for consistency, the differences were attributable primarily to the increase from 2.5% to higher macrogol 400 concentrations, whereas further increasing the concentration from 2.75% to 3% did not result in an additional effect.

Stringiness showed relatively small differences among the tested placebo FFSs, with values ranging from 28.7451 to 29.8101 mm (Table 7, Figure 5e). The highest stringiness was observed for SG-M-2.5-WI 11 (29.8101 ± 0.0723 mm), whereas the lowest value was recorded for SG-M-3-WI 11 (28.7451 ± 0.2910 mm).

Overall, the water/isopropanol ratio significantly affected the textural characteristics of the investigated FFSs, with WI 11 group FFSs generally exhibiting lower firmness, consistency, adhesiveness, and cohesiveness than the corresponding WI 21 group FFSs. Macrogol 400 concentration also significantly affected consistency, adhesiveness, and cohesiveness, whereas its interaction with solvent ratio was significant only for stringiness.

### 3.3. Characteristics of Films Prepared from DoE-Based Placebo FFSs

All placebo FFSs produced films that were readily detached from the solid substrate after drying. Films prepared from each FFS were transparent, homogeneous, and visually uniform, with no visible particles or entrapped air bubbles.

Film weights are presented in Table 8, with placebo films ranging from 274 to 322 mg. An increase in film weight was observed with increasing macrogol 400 concentration. Films prepared from the WI 21 group FFSs generally exhibited higher weights than those prepared from the WI 11 group FFSs, although these differences were less pronounced than those observed with increasing macrogol 400 concentration.

The placebo film thickness ranged from 38.7 ± 3.2 µm to 48.7 ± 3.2 µm, indicating relatively small differences among the tested formulations.

Visual assessment of cotton fiber adhesion classified all placebo film samples as moderately sticky, as evidenced by the formation of a thin layer of cotton fibers on their surfaces.

The mechanical properties of films prepared from placebo FFSs are presented in Table 8. Tensile strength values ranged from 15.59 to 21.84 MPa, elongation at break from 68.31 to 100.05%, and Young’s modulus from 63.04 to 108.51 MPa. Films prepared using the WI 11 group FFSs generally exhibited higher elongation at break and lower Young’s modulus values than those prepared using the WI 21 group FFSs, whereas tensile strength remained comparable between the two groups (Table 8). Statistical analysis showed that the isopropanol concentration did not significantly affect tensile strength (a difference of −0.110 (95% CI: −4.439, 4.218), *p* = 0.957, n = 18, Cohen’s d = 0.0262 (95% CI for Cohen’s d: −1.00, 1.05)) or elongation at break (a difference of −4.984 (95% CI: −17.228, 7.260), *p* = 0.39256, n = 18, Cohen’s d = 0.418 (95% CI for Cohen’s d: −0.63, 1.46)), whereas films prepared from the WI 11 group FFSs exhibited significantly lower Young’s modulus values than those prepared from the WI 21 group (a difference of 17.035 (95% CI: 7.922, 26.148), *p* = 0.00155, n = 18, Cohen’s d = −1.92 (95% CI for Cohen’s d: −3.26, −0.58)). Regarding the effect of macrogol 400 concentration, increasing its concentration from 2.5% to 2.75% significantly increased elongation at break and decreased Young’s modulus, whereas the increase in tensile strength was not statistically significant (a difference of 3.239 (95% CI: −3.252, 9.730), *p* = 0.426, n = 18, Cohen’s d = −0.769 (95% CI for Cohen’s d: −2.07, 0.535)). A further increase in macrogol 400 concentration to 3% did not produce any additional statistically significant improvement in the evaluated mechanical properties.

### 3.4. Characteristics of Ketoprofen-Loaded FFS and Corresponding Film

Considering all evaluated characteristics, including the rheological and textural properties of the DoE-based placebo FFSs and the mechanical properties of the corresponding films, the formulation containing 2.75% macrogol 400 and a 1:1 water/isopropanol ratio was selected as the most suitable placebo FFS for subsequent ketoprofen incorporation.

The ketoprofen-loaded FFS was a clear, colorless, and homogeneous solution without visible particles. Its pH was 4.03 (Table 5), compared with 5.60 for the corresponding placebo FFS. The ketoprofen-loaded FFS exhibited a %T of 98.71% (Table 5).

At both 20 °C and 32 °C, the ketoprofen-loaded FFS exhibited non-Newtonian pseudoplastic flow behavior (Figure 4). Compared with the corresponding placebo FFS, ketoprofen incorporation increased *k*, *η*_max_, *η*_min_, and HA, while decreasing *n* (Table 6).

The incorporation of ketoprofen increased stringiness from 29.1273 ± 0.3060 mm for the placebo FFS to 38.9007 ± 0.3494 mm following ketoprofen incorporation, whereas no differences were observed for firmness, consistency, adhesiveness, or cohesiveness (Table 7).

The ketoprofen-loaded film was colorless, transparent, smooth, and free of entrapped air bubbles, showing no visually observable differences compared with the corresponding placebo film. As presented in Table 8, the weight of the ketoprofen-loaded film (442 ± 2 mg) was higher than that of the corresponding placebo film (300 ± 2 mg), and it was classified as moderately sticky.

Ketoprofen incorporation markedly affected the mechanical properties of the resulting film (Table 8). Tensile strength, elongation at break, and Young’s modulus were all substantially reduced compared with the corresponding placebo film. The obtained ketoprofen-loaded film exhibited tensile strength below 1 MPa and elongation at break well below the desirable range for topical films (approximately 50–120%), while the Young’s modulus was 16.88 ± 1.41 MPa.

### 3.5. In Vitro Drug Release

**Figure 6:**
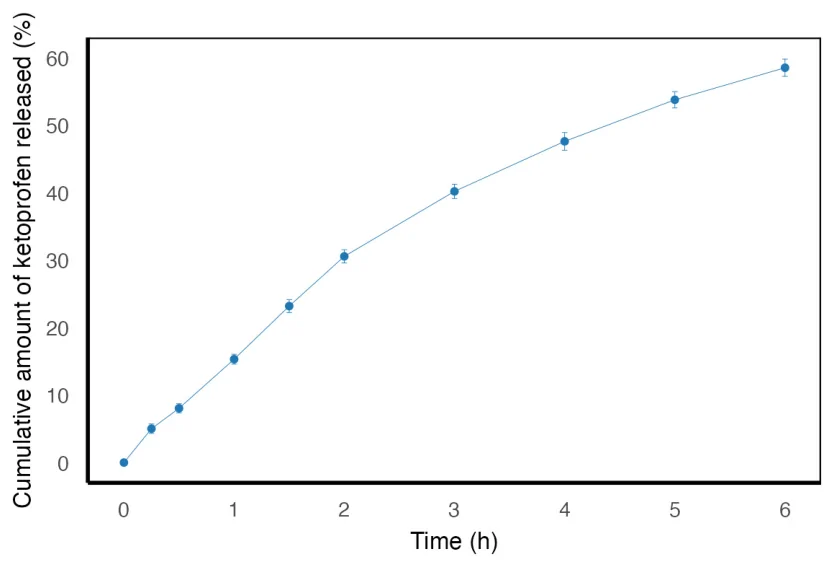
In vitro release profile of ketoprofen from the ketoprofen-loaded FFS.

Figure 6 shows the ketoprofen release profile from the FFS containing 2.75% macrogol 400 and a water/isopropanol ratio of 1:1. The total cumulative amount of ketoprofen released after 6 h was 58.52%. Model-dependent analysis of the ketoprofen release profile was performed to characterize its release kinetics and identify the model that best described the experimental data. Several kinetic models, including the first-order, Higuchi, Korsmeyer–Peppas, and Weibull models, were applied to describe the in vitro release of ketoprofen from the FFS. The corresponding model parameters and AIC values are summarized in Table 9.

Comparison of the AIC values indicated that the Weibull model provided the best fit to the ketoprofen release profile, as it yielded the lowest AIC value (−87.3318) (Table 9). The Weibull model parameters (scale parameter (*α*) and shape parameter (*β*)) were used to describe the release profile.

## 4. Discussion

The results of the factorial design provided insight into the relative contribution of the selected solvents to the mechanical performance of the placebo films. Although isopropanol concentration was expected to influence film formation due to its higher volatility and potential effects on polymer chain organization during solvent evaporation, the factorial design demonstrated that it did not significantly affect the mechanical properties of the obtained films and was therefore not retained in the final reduced response models. In contrast, macrogol 400 concentration was identified as the critical formulation factor, confirming the important role of the plasticizer in controlling film flexibility and mechanical integrity.

Since macrogol 400 was identified as the dominant formulation factor, its effect on the individual mechanical properties deserves particular consideration. The observed decrease in tensile strength (Y_1_) and Young’s modulus (Y_3_), together with the increase in elongation at break (Y_2_) with increasing macrogol 400 concentration (X_1_), can be attributed to the plasticizing effect of macrogol 400 on the hypromellose polymer matrix. At increasing plasticizer concentrations, macrogol 400 can reduce the intermolecular attractive forces between hypromellose molecules. This effect may be attributed to a possible relaxation of the interactions between hypromellose chains induced by macrogol 400 molecules, which could increase polymer chain mobility, thereby reducing film rigidity and increasing its flexibility. In addition, the incorporation of macrogol 400 into the polymer matrix can lower the glass transition temperature (Tg), facilitating molecular mobility of the polymer chains and contributing to the observed changes in the mechanical properties of the films [44]. However, the positive quadratic coefficient in the models describing tensile strength (Y_1_) and Young’s modulus (Y_3_) indicates that the magnitude of this effect is not constant across the investigated concentration range, suggesting that its relationship with tensile strength and Young’s modulus is more complex than a simple linear decrease. At higher plasticizer concentrations, changes in the balance between polymer–polymer and polymer–plasticizer interactions may contribute to the observed change in the response trend.

The concentration-dependent nature of macrogol 400 plasticization has also been reported by other authors. In films formed from FFS based on Eudragit^®^ E PO, macrogol 400 did not cause a consistent reduction in the polymer Tg with increasing its concentration. Moreover, the authors suggested that macrogol 400 acts as an effective plasticizer only within a limited concentration range and that, above a critical concentration, phase separation and plasticizer exclusion may occur [17]. Although such phenomena were not directly investigated in the present study, these findings indicate that the plasticizing efficiency of macrogol 400 may be concentration-dependent and that excess macrogol 400 may not contribute to polymer plasticization in the same manner as macrogol 400 incorporated into the polymer network. Similarly, a nonlinear relationship between macrogol 400 concentration and Tg was reported for hypromellose films, with Tg decreasing as the macrogol 400 concentration increased from 0 to 8%, although the extent of the decrease became progressively smaller at higher concentrations, indicating a tendency towards a plateau [32]. This behavior was interpreted as evidence of reduced plasticization efficiency beyond a certain macrogol 400 concentration. Together, these findings support the possibility that the effect of macrogol 400 on the mechanical properties of hypromellose films may change across the investigated concentration range, potentially contributing to the nonlinear response observed in the present study. It should also be noted that the interpretation of the model fit and predictive statistics should consider the experimental replication structure. For each formulation condition, a single FFS batch was prepared, while the three replicates represented independently cast films obtained from the same batch. Thus, the high R^2^, adjusted R^2^, and predicted R^2^ values reflect the ability of the fitted models to describe the observed variation within the investigated experimental design, but should not be interpreted as evidence of batch-to-batch reproducibility. Assessment of independent formulation batches would be required to further evaluate formulation-level reproducibility.

The established relationships between macrogol 400 concentration and mechanical characteristics of the film provided a rational basis for FFS formulation optimization. Accordingly, numerical optimization based on the predictive models generated by the factorial design was used to obtain DoE-based FFSs, from which the most suitable placebo FFS was selected. Characterization of the DoE-based placebo FFSs confirmed that, in addition to the desired mechanical properties, all formulations exhibited appropriate physicochemical characteristics for topical application. The absence of visible particles or phase separation indicated adequate solubilization of the formulation components and good physical homogeneity of the prepared FFSs. Furthermore, the obtained pH values were within the physiologically acceptable range for topical application (pH 4–6) [45], suggesting compatibility with the skin surface and a low potential for irritation. Likewise, the high %T values (>97%) confirmed the high transparency of all formulations and exceeded the previously reported threshold of 90%, indicating complete dissolution of formulation components and physical uniformity of the systems [36]. The shear-thinning behavior observed for all placebo FFSs is characteristic of polymer-based systems and is commonly attributed to the alignment of polymer chains in the direction of flow under applied shear [46]. Such behavior is advantageous for topical FFSs, as it facilitates spreading during application while maintaining sufficient viscosity at rest to reduce formulation runoff [47]. The presence of hysteresis loops confirmed the thixotropic nature of the investigated systems, indicating that their internal structure was partially disrupted during shear and gradually recovered after removal of the applied force. This reversible structural behavior is considered beneficial for topical formulations because it contributes to ease of application while allowing the formulation to regain consistency after spreading. The higher *η*_max_ and *η*_min_ values observed in the WI 21 group compared with the WI 11 group suggest that reducing the isopropanol content increased the resistance of the formulations to flow. Moreover, the influence of macrogol 400 on viscosity depended on solvent composition, indicating an interaction between the plasticizer and the solvent system in determining the rheological properties of the FFSs. The reduction in *η*_max_, *η*_min_, and *k* at 32 °C demonstrates the expected temperature-dependent decrease in formulation resistance to flow. Since this temperature corresponds to the skin surface, such behavior may facilitate spreading immediately after application. Although the formulations became less viscous at the higher temperature, they retained pseudoplastic behavior, indicating that their favorable application characteristics were preserved under conditions relevant for application to the skin. In addition to rheological properties, textural characteristics are important for evaluating the FFS performance during application and subsequent film formation. Texture profile analysis provides information on parameters such as firmness, consistency, adhesiveness, cohesiveness, and stringiness, which may influence ease of application, spreadability, and retention of the formulation on the skin [33]. Ideally, a topical FFS should exhibit sufficiently low firmness and consistency to allow easy deformation and spreading during application, while adequate adhesiveness may contribute to retaining the formulation at the application site long enough to enable solvent evaporation and subsequent film formation [34]. Therefore, the combined evaluation of these parameters provides valuable insight into the overall application performance of the investigated formulations. The relatively low firmness values obtained for all DoE-based placebo FFSs indicate low resistance to deformation, suggesting favorable handling and application characteristics. The consistently higher firmness and consistency of WI 21 group FFSs were in agreement with the rheological findings, indicating greater resistance to deformation during handling and spreading. The higher adhesiveness and cohesiveness observed for WI 21 group FFSs indicate stronger interactions with the contacting surface and greater internal structural integrity. From a practical perspective, increased adhesiveness may improve formulation retention at the application site immediately after administration. However, excessively high adhesiveness may adversely affect the sensory properties of the formulation and reduce patient comfort during application. The effect of macrogol 400 concentration on consistency, adhesiveness, and cohesiveness was most pronounced when its concentration increased from 2.5% to 2.75%, whereas further increasing the concentration to 3% did not produce additional statistically significant improvements. These findings suggest that the influence of macrogol 400 on these textural properties reaches a plateau within the investigated concentration range. Unlike the other textural parameters, the significant interaction observed for stringiness indicates that the influence of macrogol 400 depended on the water/isopropanol ratio. Consequently, the effect of the plasticizer on stringiness cannot be interpreted independently of the solvent composition. Overall, these findings demonstrate that the water/isopropanol ratio was the primary determinant of the textural characteristics of the investigated FFSs, whereas the effect of macrogol 400 varied among the evaluated textural parameters and became limited above approximately 2.75%. Therefore, increasing the plasticizer concentration beyond this level is unlikely to provide meaningful improvements in textural performance while potentially introducing unnecessary changes in FFS and film properties.

Since the performance of FFSs depends not only on the properties in the liquid state but also on the characteristics of the film formed after solvent evaporation, the obtained films were further evaluated. The transparent, homogeneous, and visually uniform appearance of all films formed from DoE-based placebo FFSs, together with the absence of visible particles or entrapped air bubbles, indicated good compatibility of the formulation components and their homogeneous distribution throughout the polymeric film matrix. The increase in film weight with increasing macrogol 400 concentration can be attributed to the higher amount of non-volatile component incorporated into the formulation, with macrogol 400 being retained within the polymeric film matrix after solvent evaporation. Although films prepared from the WI 21 group FFSs were generally heavier than those from the WI 11 group, the effect of solvent composition on film weight was less pronounced than that of the plasticizer concentration. Among the evaluated film characteristics, mechanical properties are of particular importance, as they directly influence film integrity and flexibility on the skin. Sufficient tensile strength is required to preserve film integrity, whereas high elongation at break together with a relatively low Young’s modulus reflects greater flexibility, allowing the film to accommodate skin movements while minimizing the risk of cracking. The obtained results demonstrate that both isopropanol and macrogol 400 concentration influenced the mechanical behavior of the films. In particular, the lower Young’s modulus and higher elongation at break observed for films prepared from the WI 11 group FFSs indicate more flexible films compared with the corresponding WI 21 group formulations. Furthermore, increasing the macrogol 400 concentration from 2.5% to 2.75% improved the overall mechanical profile of the films, whereas a further increase to 3% did not provide additional benefits.

The most suitable placebo FFS for ketoprofen incorporation was selected based on the evaluation of the characteristics of the investigated DoE-based placebo FFSs and their corresponding films. Increasing the macrogol 400 concentration from 2.5% to 2.75% significantly affected the rheological and textural characteristics of the placebo FFSs, whereas a further increase to 3% did not result in statistically significant changes. More importantly, the increase in macrogol 400 concentration from 2.5% to 2.75% resulted in improved mechanical properties of the corresponding films, as evidenced by increased tensile strength and elongation at break and decreased Young’s modulus. Further increasing the macrogol 400 concentration to 3% did not provide additional improvement, as tensile strength even decreased, while changes in elongation at break and Young’s modulus were not statistically significant. Within the investigated range, a higher proportion of the volatile solvent was selected as a practical formulation-development consideration, since sufficiently rapid solvent evaporation and film formation are relevant performance requirements for topical FFSs. Furthermore, the water/isopropanol ratio 1:1 provided more favorable characteristics than the 2:1 ratio, including lower viscosity of the FFSs and lower Young’s modulus of the corresponding films, suggesting easier application together with improved film flexibility. Based on these findings, the FFS containing 2.75% macrogol 400 and a water/isopropanol mixture at a 1:1 ratio was considered the most suitable placebo FFS for ketoprofen incorporation.

Given its low aqueous solubility (0.051 mg/mL in pure water at 22 °C) [2] and pH-dependent solubility [48], achieving complete dissolution of ketoprofen in the FFS was of particular importance. The incorporation of ketoprofen resulted in no significant changes in the physicochemical characteristics of the FFS. The clear appearance, high %T, and absence of visible particles in the ketoprofen-loaded FFS were consistent with the absence of visible drug precipitation and suggested complete dissolution of ketoprofen. However, confirmation of molecular-level solubilization would require additional characterization, such as microscopic analysis, differential scanning calorimetry (DSC), X-ray powder diffraction (XRPD), or another appropriate solid-state or compatibility characterization. As expected, ketoprofen incorporation reduced the formulation pH because of the acidic nature of the drug (pKa ≈ 4.45) [2]. Nevertheless, the obtained pH remained within the physiologically acceptable range for skin application [45]. In addition, at this pH ketoprofen is likely predominantly present in its non-ionized form, which is generally considered more favorable for passive diffusion across the stratum corneum [49]. Although ketoprofen incorporation increased the *η*_max_, *η*_min_, *k*, and HA while reducing the *n*, the FFS retained its pseudoplastic behavior at both investigated temperatures. The observed shear-thinning behavior is advantageous for topical FFSs because the formulation becomes less viscous during spreading while recovering viscosity after application, thereby promoting retention at the application site during solvent evaporation and film formation. Among the investigated textural properties, ketoprofen significantly affected only stringiness, while firmness, consistency, adhesiveness, and cohesiveness remained unchanged. The marked increase in stringiness may be caused by ketoprofen interactions with the hypromellose network that promoted polymer chain extension during probe withdrawal. Since the remaining textural properties were unaffected, ketoprofen incorporation appears to have had only a limited influence on the structural characteristics of the FFS. In contrast, ketoprofen incorporation had a much more pronounced influence on the properties of the film formed from the FFS, particularly its mechanical performance. Although the ketoprofen-loaded film remained visually uniform and exhibited acceptable stiffness, as reflected by the Young’s modulus, it showed a pronounced reduction in tensile strength and elongation at break. These findings indicate that the drug-loaded film became substantially less resistant to mechanical stress and would therefore be expected to have a lower capacity to accommodate deformation following its formation on the skin. While the experimentally selected placebo FFS exhibited favorable mechanical performance, incorporation of ketoprofen may have modified polymer-chain interactions and the microstructure of the film, potentially contributing to the observed reduction in mechanical integrity. However, confirmation of these potential drug–polymer interactions would require additional physicochemical characterization, such as Fourier-transform infrared spectroscopy (FTIR), DSC, and/or XRPD. This observation emphasizes that optimization based solely on placebo formulations may not fully predict the behavior of the final drug-loaded system.

Although the primary focus of this work was on the applicative characteristics of the FFSs, the in vitro release of ketoprofen was also evaluated to determine the extent to which the formulations could release the incorporated drug. The cumulative amount of ketoprofen released from ketoprofen-loaded FFS after 6 h was 58.52%. This release was lower than that previously reported for another optimized hypromellose-based ketoprofen FFS (82.66% after 6 h), but remained substantially higher than the release achieved from commercially available ketoprofen gels (approximately 34.5%), which were tested in our previous study under the same experimental conditions [15]. Other studies on ketoprofen-containing FFSs have likewise reported relatively limited drug release. For example, approximately 35% of the loaded ketoprofen, corresponding to about 70 μg/cm^2^, was released from a Eudragit E100-based FFS, with the maximum release reached after 2 h [50]. The Weibull model was selected as the most suitable model for describing ketoprofen release from the FFS due to its lowest AIC value, indicating the best agreement with the experimental data among the investigated kinetic models. The α value was 6.6327, corresponding to the model-estimated time in hours required to achieve 63.2% drug release. The *β* value was 0.8945, indicating a decelerating release profile characterized by a relatively faster release at earlier time points followed by a gradual decrease in the release rate over time. Although the Weibull model is empirical, the *β* parameter has been used to characterize different types of drug release profiles and has been associated with different release mechanisms [51]. According to this interpretation, *β* values between 0.75 and 1 suggest a combined release mechanism involving Fickian diffusion and swelling-controlled transport [51]. Therefore, the obtained *β* value indicates the possible contribution of both diffusion and polymer swelling/relaxation processes during ketoprofen release from FFS. Since the Weibull model does not directly provide mechanistic information, the Korsmeyer–Peppas model was also considered to provide insight into the drug release mechanism. The obtained *n* value of 0.7009 suggests anomalous (non-Fickian) transport, further supporting the involvement of more than one process in the release mechanism. The release behavior observed in the present study is also consistent with the complex release mechanisms previously described for hypromellose-based FFSs. In a study by Labella-Lorite et al., hypromellose-containing FFSs exhibited anomalous transport of mometasone furoate according to the Korsmeyer–Peppas model, with release attributed to the combined effects of drug diffusion and polymer swelling, chain disentanglement, and relaxation following solvent penetration into the polymeric matrix [14]. The observed release behavior may be attributed to the complex dynamics of the hypromellose-based FFS upon contact with the release medium, where polymer hydration, chain relaxation, and drug diffusion may occur simultaneously. Nevertheless, this finding should be interpreted with caution, as definitive assignment of the release mechanism would require additional mechanistic studies.

It is important to emphasize that the primary aim of the present study was to investigate the effect of solvent composition, particularly macrogol 400 and isopropanol, on the mechanical characteristics of hypromellose-based films obtained from FFSs, while also considering relevant applicative properties of the FFSs. The subsequent incorporation of ketoprofen was used to provide an initial characterization of the selected formulation in terms of its physicochemical, mechanical, textural, and in vitro drug-release properties. Further development of this system requires additional investigation of both the ketoprofen-loaded FFS and the resulting film, particularly to elucidate how ketoprofen incorporation affects the structural and physicochemical characteristics of the film matrix. In addition, studies addressing drug permeation through the skin, as well as the behavior and performance of the film under in vivo conditions, are warranted to further evaluate the potential of the developed FFS as a topical dosage form.

## 5. Conclusions

A hypromellose-based placebo FFS was developed using a 3^2^ full factorial design-based approach, identifying macrogol 400 as the key variable governing film mechanical properties. The factorial design revealed a quadratic relationship between macrogol 400 concentration and tensile strength as well as macrogol 400 concentration and Young’s modulus, whereas elongation at break showed a linear dependence. Characterization of the DoE-based placebo FFSs confirmed the positive effect of macrogol 400 content up to 2.75% on film mechanical properties, while further increase did not provide additional improvements. The water/isopropanol ratio primarily influenced FFS rheological and textural characteristics, with a limited effect on film mechanical properties, affecting only Young’s modulus. The experimentally selected placebo FFS exhibited suitable characteristics for topical application and provided an appropriate vehicle for ketoprofen incorporation. The ketoprofen-loaded FFS demonstrated a higher extent of drug release than previously reported for commercial ketoprofen gels. Although the drug presence caused changes in film mechanical properties, which could indicate reduced capacity to accommodate deformation, the ketoprofen-loaded film demonstrated preserved integrity and quality attributes (transparency, low weight, and moderate stickiness) that are relevant to patient acceptability. The developed FFS represents a promising topical dosage form for non-steroidal anti-inflammatory drugs and warrants further investigation, particularly with respect to additional characterization of the drug-loaded film, skin retention, and in vivo performance.

## Figures and Tables

**Table 1:** Levels and the experimental conditions for a 3^2^ factorial design.

Factor	Designation	Low Level (–1)	Medium Level (0)	High Level (+1)
X_1_	Concentration of macrogol 400 (% *w*/*w*)	0	2.5	5
X_2_	Concentration of isopropanol (% *w*/*w*)	25	33	50

**Table 2:** Experimental runs for a 3^2^ factorial design.

FFS Sample	Factor X_1_	Factor X_2_	Response Y_1_	Response Y_2_	Response Y_3_
	Concentration of Macrogol 400 (% *w*/*w*)	Concentration of Isopropanol (% *w*/*w*)	Tensile Strength ± S.D. (MPa) ^a^	Elongation at Break ± S.D. (%) ^a^	Young’s Modulus ± S.D. (MPa) ^a^
SG-M-0-WI 31	0 (–1)	25 (–1)	59.37 ± 4.74 ^b^	3.58 ± 2.38 ^b^	3246.09 ± 182.97 ^b^
SG-M-2.5-WI 31	2.5 (0)	25 (–1)	20.41 ± 2.96 ^b^	74.38 ± 4.69 ^b^	117.65 ± 14.91 ^b^
SG-M-5-WI 31	5 (+1)	25 (–1)	7.17 ± 0.32 ^b^	55.17 ± 3.70 ^b^	52.82 ± 11.48 ^b^
SG-M-0-WI 21	0 (–1)	33 (0)	65.74 ± 4.21	7.78 ± 1.52	3297.59 ± 178.17
SG-M-2.5-WI 21	2.5 (0)	33 (0)	15.59 ± 5.56	68.31 ± 17.51	108.51 ± 3.42
SG-M-5-WI 21	5 (+1)	33 (0)	16.17 ± 3.43	123.52 ± 8.55	34.66 ± 16.79
SG-M-0-WI 11	0 (–1)	50 (+1)	63.72 ± 4.80 ^b^	3.26 ± 0.33 ^b^	3037.21 ± 284.03 ^b^
SG-M-2.5-WI 11	2.5 (0)	50 (+1)	19.18 ± 3.66 ^b^	77.82 ± 5.73 ^b^	96.78 ± 8.72 ^b^
SG-M-5-WI 11	5 (+1)	50 (+1)	12.42 ± 1.71 ^b^	91.49 ± 8.39 ^b^	51.87 ± 5.17 ^b^

^a^ Values are expressed as mean ± SD of three independently cast films prepared from one FFS batch for each experimental condition. ^b^ Data previously reported in [15].

**Table 3:** Composition of placebo FFSs based on a 3^2^ full factorial design.

Ingredient	FFSs with Water/Isopropanol Mixture 2:1 (WI 21 Group) (% *w*/*w*)			FFSs with water/Isopropanol Mixture 1:1 (WI 11 Group) (% *w*/*w*)		
	SG-M-2.5-WI 21	SG-M-2.75-WI 21	SG-M-3-WI 21	SG-M-2.5-WI 11	SG-M-2.75-WI 11	SG-M-3-WI 11
Hypromellose	2.00	2.00	2.00	2.00	2.00	2.00
Macrogol 400	2.50	2.75	3.00	2.50	2.75	3.00
Purified water	63.67	63.50	63.33	47.75	47.625	47.50
Isopropanol	31.83	31.75	31.67	47.75	47.625	47.50

**Table 4:** Model-predicted and experimentally observed mechanical responses of the experimentally selected placebo FFS.

Response	Predicted Value	95% Prediction Interval	Observed Value	Relative Prediction Error
Tensile strength	16.03 MPa	6.09–25.98 MPa	19.41 ± 2.43 MPa	17.4%
Elongation at break	60.40%	2.64–118.17%	95.69 ± 8.49%	36.9%
Young’s modulus	≈66.30 MPa	31.23–112.35 MPa	66.14 ± 18.09 MPa	0.24%

**Table 5:** pH and transmittance (%T) of DoE-based placebo FFSs and ketoprofen-loaded FFS (designated with the suffix “-KET”).

FFS Sample	pH	%T (%)
SG-M-2.5-WI 21	4.98	97.71
SG-M-2.75-WI 21	4.99	98.02
SG-M-3-WI 21	5.01	97.89
SG-M-2.5-WI 11	5.57	98.77
SG-M-2.75-WI 11	5.60	97.77
SG-M-3-WI 11	5.62	97.67
SG-M-2.75-WI 11-KET	4.03	98.71

**Table 6:** Rheological parameters (flow behavior index (*n*), consistency index (*k*), maximum apparent viscosity (*η*_max_) (at 10.5 s^−1^), minimum apparent viscosity (*η*_min_) (at 200 s^−1^) and hysteresis area (HA)) of DoE-based placebo FFSs and ketoprofen-loaded FFS (designated with the suffix “-KET”) at 20 °C and 32 °C.

FFS Sample	T (°C)	*n* ± S.D.	*k* ± S.D. ((Pa·s)^n^)	*η*_max_ ± S.D. (mPa·s)	*η*_min_ ± S.D. (mPa·s)	HA ± S.D. (Pa/s)
SG-M-2.5-WI 21	20	0.4721 ± 0.0010	28.4390 ± 0.5017	7316.67 ± 120.55	1660.00 ± 26.46	740.5000 ± 73.5001
	32	0.5487 ± 0.0041	14.3459 ± 0.2888	4363.33 ± 80.83	1250.00 ± 0.00	903.0033 ± 22.0086
SG-M-2.75-WI 21	20	0.4617 ± 0.0027	30.4439 ± 1.0955	7743.33 ± 241.32	1686.67 ± 40.41	639.2250 ± 109.3210
	32	0.5587 ± 0.0036	13.1349 ± 0.2722	4063.33 ± 75.05	1203.33 ± 5.77	718.6300 ± 40.0017
SG-M-3-WI 21	20	0.4680 ± 0.0025	29.5630 ± 0.4512	7556.67 ± 80.21	1686.67 ± 20.82	630.3717 ± 187.4263
	32	0.5534 ± 0.0036	13.7636 ± 0.2818	4146.25 ± 75.78	1226.61 ± 5.87	895.7904 ± 41.6465
SG-M-2.5-WI 11	20	0.5018 ± 0.0053	19.8815 ± 0.3994	5506.67 ± 55.08	1353.33 ± 11.55	473.1567 ± 83.7121
	32	0.5890 ± 0.0027	9.4209 ± 0.1742	3223.33 ± 57.73	1013.33 ± 5.77	617.8617 ± 68.6700
SG-M-2.75-WI 11	20	0.4799 ± 0.0013	22.9981 ± 0.3241	5986.67 ± 75.05	1403.33 ± 11.55	1060.0417 ± 69.6732
	32	0.5570 ± 0.0027	11.5320 ± 0.1686	3573.33 ± 120.55	1053.33 ± 5.77	1107.9133 ± 164.6115
SG-M-3-WI 11	20	0.4778 ± 0.0004	24.0512 ± 0.2211	6310.00 ± 45.83	1453.33 ± 11.55	938.3383 ± 26.8261
	32	0.5538 ± 0.0045	12.0443 ± 0.3302	3683.33 ± 109.70	1083.33 ± 5.77	1156.8867 ± 29.7538
SG-M-2.75-WI 11-KET	20	0.4681 ± 0.0045	26.2161 ± 1.0549	6623.33 ± 174.74	1503.33 ± 30.55	1669.5933 ± 453.5341
	32	0.5247 ± 0.0032	14.9685 ± 0.4165	4140.00 ± 34.64	1156.67 ± 11.55	2188.8450 ± 355.3389

**Table 7:** Texture profile analysis parameters (firmness, consistency, adhesiveness, cohesiveness, and stringiness) of DoE-based placebo FFSs and ketoprofen-loaded FFS (designated with the suffix “-KET”).

FFS Sample	Firmness (N)	Consistency (mJ)	Adhesiveness (mJ)	Cohesiveness (N)	Stringiness (mm)
SG-M-2.5-WI 21	0.2684 ± 0.0175	2.9200 ± 0.1711	1.2324 ± 0.1317	0.0566 ± 0.0084	29.4899 ± 0.0675
SG-M-2.75-WI 21	0.2885 ± 0.0209	3.1288 ± 0.1919	1.4830 ± 0.1696	0.0755 ± 0.0120	29.5778 ± 0.0397
SG-M-3-WI 21	0.2904 ± 0.0171	3.1378 ± 0.1653	1.5130 ± 0.1321	0.0787 ± 0.0095	29.5895 ± 0.0321
SG-M-2.5-WI 11	0.2724 ± 0.0629	2.2551 ± 0.1676	0.5072 ± 0.0832	0.0337 ± 0.0039	29.8101 ± 0.0723
SG-M-2.75-WI 11	0.2376 ± 0.0209	2.5858 ± 0.1889	0.8461 ± 0.1851	0.0383 ± 0.0042	29.1273 ± 0.3060
SG-M-3-WI 11	0.2177 ± 0.0144	2.4441 ± 0.1546	0.8581 ± 0.1164	0.0408 ± 0.0030	28.7451 ± 0.2910
SG-M-2.75-WI 11-KET	0.2259 ± 0.0159	2.4428 ± 0.1107	0.9588 ± 0.1239	0.0382 ± 0.0018	38.9007 ± 0.3494

**Table 8:** Film weight (FW), film thickness (FT), tensile strength (TS), elongation at break (EB), and Young’s modulus (YM) of films formed from DoE-based placebo FFSs and ketoprofen-loaded FFS (the films are labeled with the same labels of the corresponding FFSs from which they were obtained).

Film Sample	FW ± S.D. (mg)	FT ± S.D. (µm)	TS ± S.D. (MPa)	EB ± S.D. (%)	YM ± S.D. (MPa)
SG-M-2.5-WI 21	280 ± 3	38.7 ± 3.2	15.59 ± 5.56	68.31 ± 17.51	108.51 ± 3.42
SG-M-2.75-WI 21	304 ± 1	40.3 ± 1.5	21.84 ± 3.59	94.56 ± 7.86	93.16 ± 1.76
SG-M-3-WI 21	322 ± 1	43.3 ± 2.5	19.94 ± 4.41	96.24 ± 15.09	75.39 ± 2.57
SG-M-2.5-WI 11	274 ± 2	43.3 ± 2.9	19.18 ± 3.66	77.82 ± 5.73	96.78 ± 8.72
SG-M-2.75-WI 11	300 ± 2	46.7 ± 0.6	19.41 ± 2.43	95.69 ± 8.49	66.14 ± 18.09
SG-M-3-WI 11	319 ± 1	48.7 ± 3.2	19.12 ± 4.90	100.05 ± 11.06	63.04 ± 7.14
SG-M-2.75-WI 11-KET	442 ± 2	85.0 ± 9.1	0.99 ± 0.08	12.60 ± 1.26	16.88 ± 1.41

**Table 9:** Kinetic models applied to in vitro ketoprofen release from ketoprofen-loaded FFS, including AIC values, coefficient of determination (R^2^), and model parameters.

Model	AIC Value	R^2^	Parameter	Value
1st-order	−78.7111	0.9922	*K* (h^−1^)	0.1609
Higuchi	−59.9120	0.9492	*K_H_* (h^−1/2^)	0.2252
Korsmeyer–Peppas	−75.5389	0.9913	*K_K-P_* (h^−n^)	0.1741
			*n*	0.7009
Weibull	−87.3318	0.9973	*α*	6.6327
			*β*	0.8945

## Data Availability

The data presented in this study are available on request from the corresponding author.

## Supplementary Materials

The following supporting information can be downloaded at: https://www.mdpi.com/article/10.3390/pharmaceutics18101249/s1. Table S1. Model adequacy and predictive-performance statistics; Table S2. ANOVA and regression coefficients for the reduced quadratic model describing tensile strength; Table S3. ANOVA and regression coefficients for the reduced linear model describing elongation at break; Table S4. ANOVA and regression coefficients for the reduced quadratic model describing square-root transformed Young’s modulus; Figure S1. Predicted versus experimentally observed values for (a) tensile strength, (b) elongation at break, and (c) Young’s modulus. The diagonal line represents perfect agreement between predicted and observed values. Young’s modulus predictions are presented on the original scale after back-transformation of the square-root model; Figure S2. Internally studentized residuals versus predicted values for (a) tensile strength, (b) elongation at break, and (c) square-root transformed Young’s modulus. Dashed horizontal lines indicate residual values of ±3; Figure S3. Normal Q–Q plots of internally studentized residuals for (a) tensile strength, (b) elongation at break, and (c) square-root transformed Young’s modulus.

## Abbreviations

The following abbreviations are used in this manuscript:

AIC	Akaike information criterion
DoE	Design of experiments
EB	Elongation at break
FFSs	Film-forming solutions
FW	Film weight
HA	Hysteresis area
ISO	International organization for standardization
KET	Ketoprofen
M	Macrogol 400
NSAIDs	Non-steroidal anti-inflammatory drugs
Ph. Eur.	European Pharmacopoeia
SG	Sigma hypromellose
TS	Tensile strength
Tg	Glass transition temperature
USP	United States Pharmacopeia
WI 11	Water/isopropanol ratio 1:1
WI 21	Water/isopropanol ratio 2:1
WI 31	Water/isopropanol ratio 3:1
YM	Young’s modulus
%T	Transmittance
