# Supplementary material for: Understanding the Role of Solvents in Hypromellose-Based Film-Forming Solutions for Topical Administration of Ketoprofen Through a Design of Experiments Approach
Source: Pharmaceutics. 2026 Oct 1;18(10):1249. doi: 10.3390/pharmaceutics18101249 (PMC13708587; doi:10.3390/pharmaceutics18101249)
Supplement: Supplementary file 1 [file pharmaceutics-18-01249-s001.zip › pharmaceutics-4571477-supplementary.pdf]

Table S1. Model adequacy and predictive-performance statistics.

Response	Model	Transform	F	p	R^2	Adj R^2	Pred R^2	CV (%)	Adeq Precision	Lack-of-fit
Tensile strength	Reduced quadratic	None	186.16	<0.0001	0.9841	0.9789	0.9643	11.34	25.061	Not estimable*
Elongation at break	Reduced linear	None	20.30	0.0028	0.7436	0.7069	0.5507	41.25	7.803	Not estimable*
Young's modulus	Reduced quadratic	Square root	2922.65	<0.0001	0.9990	0.9986	0.9977	3.62	96.933	Not estimable*

*Independent FFS-batch replicates were not included at identical design points; therefore, batch-level pure error and formal lack-of-fit could not be estimated.

Table S2. ANOVA and regression coefficients for the reduced quadratic model describing tensile strength.

Parameter	Estimate / Sum of squares	df	SE / Mean square	F-value	p-value	95% CI
ANOVA: Model	4629.99	2	2314.99	186.16	<0.0001	—
ANOVA: Macrogol 400 concentration (X_1)	3905.07	1	3905.07	314.03	<0.0001	—
ANOVA: X_1^2	724.92	1	724.92	58.29	0.0003	—
ANOVA: Residual	74.61	6	12.44	—	—	—
ANOVA: Corrected total	4704.60	8	—	—	—	—
Coefficient: Intercept	18.39	—	2.04	—	—	13.41 to 23.38
Coefficient: X_1	−25.51	—	1.44	—	—	−29.03 to −21.99
Coefficient: X_1^2	19.04	—	2.49	—	—	12.94 to 25.14

Final equation in coded factors: $TS = 18.39 - 25.51X_1 + 19.04X_1^2$

Table S3. ANOVA and regression coefficients for the reduced linear model describing elongation at break.

Parameter	Estimate / Sum of squares	df	SE / Mean square	F-value	p-value	95% CI
ANOVA: Model	10885.15	1	10885.15	20.30	0.0028	—
ANOVA: Macrogol 400 concentration (X_1)	10885.15	1	10885.15	20.30	0.0028	—
ANOVA: Residual	3753.84	7	536.26	—	—	—
ANOVA: Corrected total	14638.99	8	—	—	—	—
Coefficient: Intercept	56.15	—	7.72	—	—	37.89 to 74.40
Coefficient: X_1	42.59	—	9.45	—	—	20.24 to 64.95

Final equation in coded factors: $EB = 56.15 + 42.59X_1$

Table S4. ANOVA and regression coefficients for the reduced quadratic model describing square-root transformed Young's modulus.

Parameter	Estimate / Sum of squares	df	SE / Mean square	F-value	p-value	95% CI
ANOVA: Model	4613.23	2	2306.61	2922.65	<0.0001	—
ANOVA: Macrogol 400 concentration (X_1)	3707.76	1	3707.76	4698.01	<0.0001	—
ANOVA: X_1^2	905.46	1	905.46	1147.29	<0.0001	—
ANOVA: Residual	4.74	6	0.79	—	—	—
ANOVA: Corrected total	4617.96	8	—	—	—	—
Coefficient: Intercept	10.37	—	0.51	—	—	9.11 to 11.62
Coefficient: X_1	−24.86	—	0.36	—	—	−25.75 to −23.97
Coefficient: X_1^2	21.28	—	0.63	—	—	19.74 to 22.81

Young's modulus was square-root transformed prior to model fitting. Final equation in coded factors: $\sqrt{YM} = 10.37 - 24.86X_1 + 21.28X_1^2$

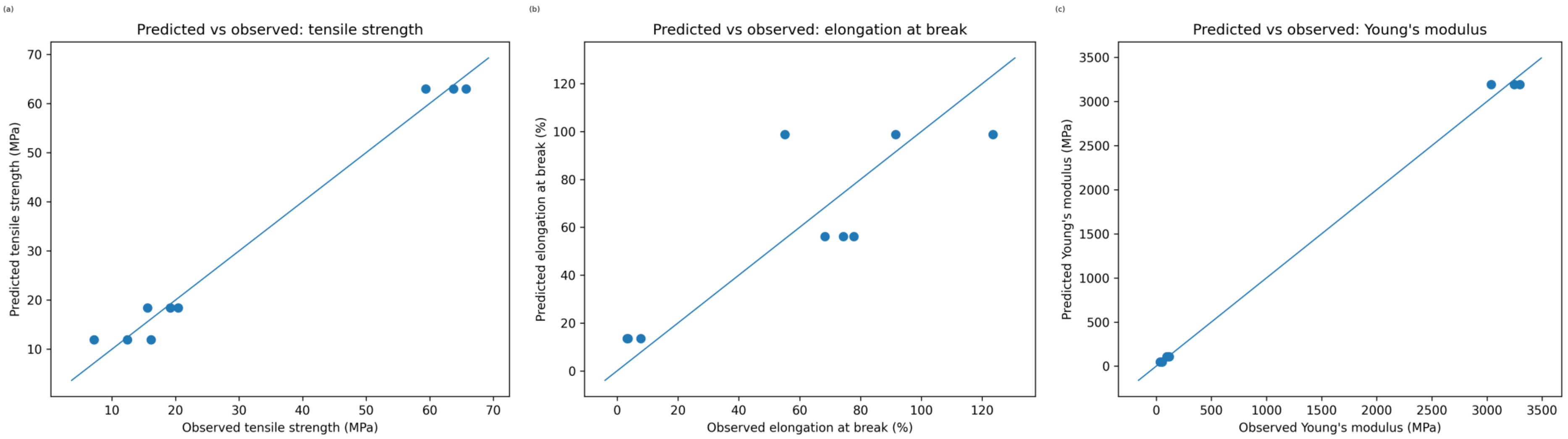

Figure S1. Predicted versus experimentally observed values for (a) tensile strength, (b) elongation at break, and (c) Young's modulus. The diagonal line represents perfect agreement between predicted and observed values. Young's modulus predictions are presented on the original scale after back-transformation of the square-root model.

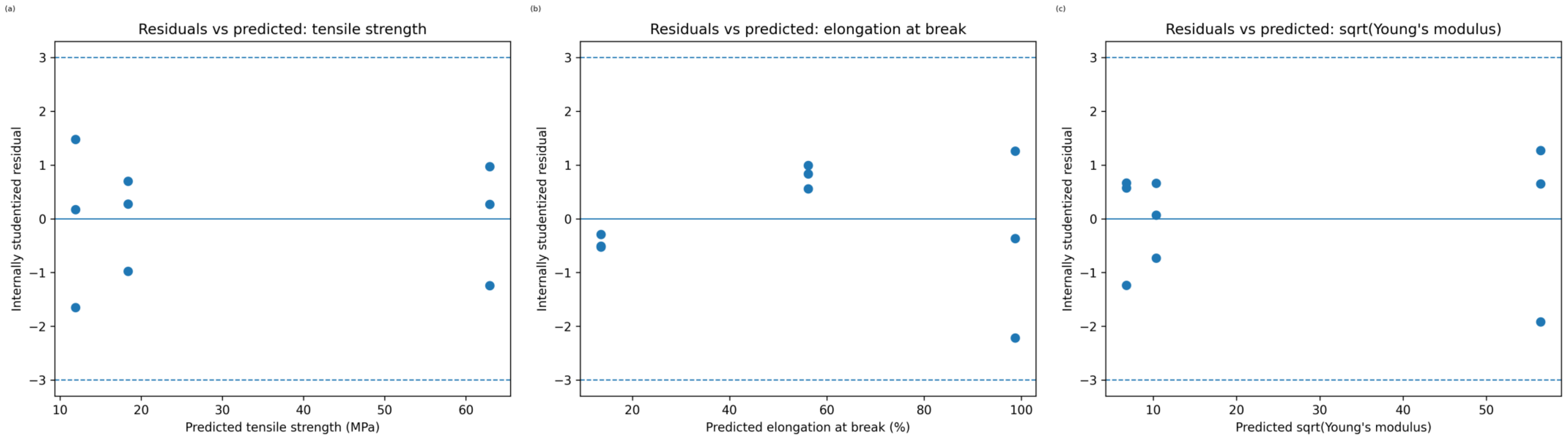

Figure S2. Internally studentized residuals versus predicted values for (a) tensile strength, (b) elongation at break, and (c) square-root transformed Young's modulus. Dashed horizontal lines indicate residual values of ±3.

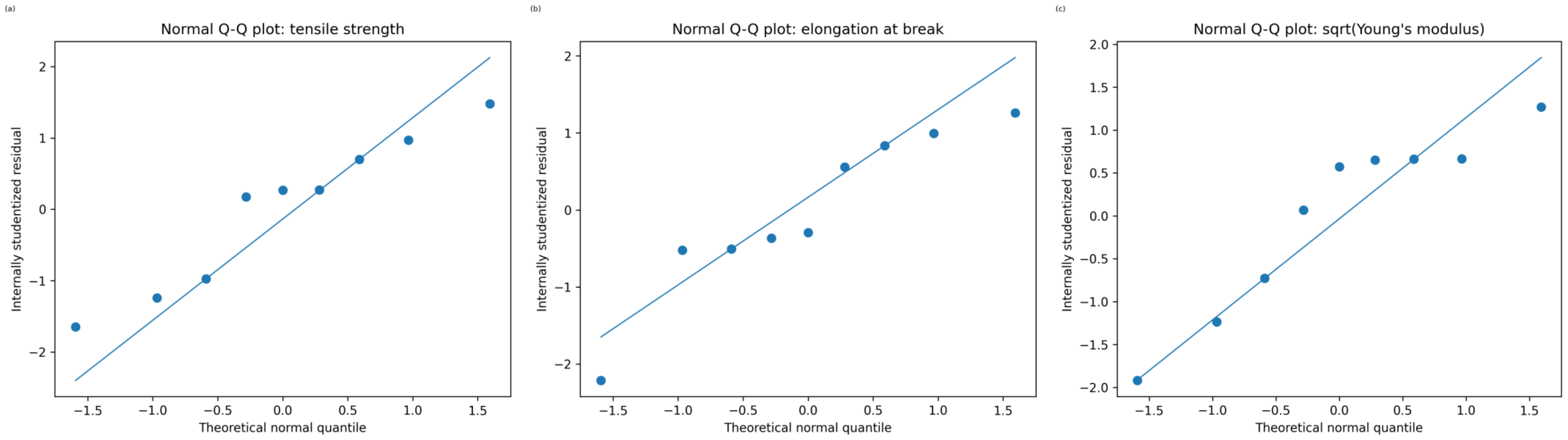

Figure S3. Normal Q–Q plots of internally studentized residuals for (a) tensile strength, (b) elongation at break, and (c) square-root transformed Young's modulus.
